# Implications of differential peroxyl radical-induced inactivation of glucose 6-phosphate dehydrogenase and 6-phosphogluconate dehydrogenase for the pentose phosphate pathway

**DOI:** 10.1038/s41598-022-25474-x

**Published:** 2022-12-07

**Authors:** Juan Sebastián Reyes, Eduardo Fuentes-Lemus, Juan David Figueroa, Javier Rojas, Angélica Fierro, Felipe Arenas, Per M. Hägglund, Michael J. Davies, Camilo López-Alarcón

**Affiliations:** 1 Departamento de Química Física, Facultad de Química y de Farmacia, Pontificia Universidad Católica de Chile, Santiago, Chile; 2 Department of Biomedical Sciences, Panum Institute, University of Copenhagen, Copenhagen, Denmark; 3 Departamento de Química Orgánica, Facultad de Química y de Farmacia, Pontificia Universidad Católica de Chile, Santiago, Chile; 4 Departamento de Biología, Facultad de Química y Biología, Universidad de Santiago de Chile, Santiago, Chile

**Keywords:** Chemical biology, Proteins

## Abstract

*Escherichia coli* glucose-6-phosphate dehydrogenase (G6PDH) and 6-phosphogluconate dehydrogenase (6PGDH) are key enzymes of the pentose phosphate pathway, responsible for the NADPH production in cells. We investigated modification of both enzymes mediated by peroxyl radicals (ROO^·^) to determine their respective susceptibilities to and mechanisms of oxidation. G6PDH and 6PGDH were incubated with AAPH (2,2′-azobis(2-methylpropionamidine)dihydrochloride), which was employed as ROO^·^ source. The enzymatic activities of both enzymes were determined by NADPH release, with oxidative modifications examined by electrophoresis and liquid chromatography (LC) with fluorescence and mass (MS) detection. The activity of G6PDH decreased up to 62.0 ± 15.0% after 180 min incubation with 100 mM AAPH, whilst almost total inactivation of 6PGDH was determined under the same conditions. Although both proteins contain abundant Tyr (particularly 6PGDH), these residues were minimally affected by ROO^·^, with Trp and Met being major targets. LC–MS and in silico analysis showed that the modification sites of G6PDH are distant to the active site, consistent with a dispersed distribution of modifications, and inactivation resulting from oxidation of multiple Trp and Met residues. In contrast, the sites of oxidation detected on 6PGDH are located close to its catalytic site indicating a more localized oxidation, and a consequent high susceptibility to ROO^·^-mediated inactivation.

## Introduction

In biological systems proteins are the major targets for oxidants^1^. Highly reactive species, such as hydroxyl radicals, HO^·^, induce diverse modifications at the side chain of all amino acids, as well as at the peptide backbone^1,2^. In contrast, moderately reactive oxidants, such as peroxyl radicals, ROO^·^, which are commonly generated in biological milieus, mediate selective modifications at the side chains of a few amino acids, including tryptophan (Trp), tyrosine (Tyr), methionine (Met), cysteine (Cys), and to a less extent, histidine (His)^3^. For a particular protein, the extent of oxidation of these residues is determined by a number of factors including the total content of each residue, exposure to the bulk solvent, protein conformations, and secondary reactions triggered by reactive intermediates^1^. As a consequence, the pattern of protein oxidation is attributed to multiple factors, making it difficult to predict, for a particular protein, the oxidative pathways, the extent and distribution of oxidation products, and the related biological consequences.

In eukaryotic and prokaryotic cells, ROO^·^ are generated during normal metabolism, as well as under oxidative stress conditions (e.g. those triggered by exogenous oxidants)^4–6^. Reactions of ROO^·^ towards proteins in such systems are likely to affect protein functionality, which could be of particular importance and significance for proteins involved in cellular metabolism. In this context, oxidation of enzymes critical to cell survival, such as those involved in the pentose phosphate pathway (PPP) are likely to be of importance. The PPP is key for metabolism and viability of eukaryotic and prokaryotic cells, playing a pivotal role in both the production of substrates for nucleotide synthesis, and the generation of reduced nicotinamide-adenine dinucleotide phosphate (NADPH), a cofactor required for fatty acid synthesis and the activity of antioxidant enzymes (e.g. glutathione peroxidases, thioredoxins, and enzymes dependent on these species such as peroxiredoxins)^7,8^. The relevance of the PPP for cells is reflected by its close association with cell proliferation, and the maintenance of cellular redox homeostasis.

**Figure 1:**
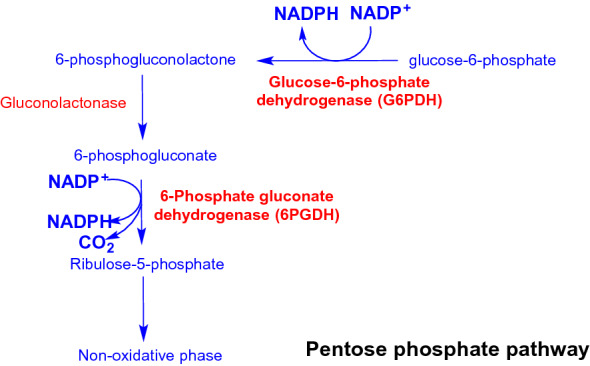
General scheme of the reactions of the pentose phosphate pathway (PPP) with the enzymes of the oxidative phase highlighted in red.

The PPP comprises two phases, the first being a non-reversible oxidative branch, in which, glucose-6-phosphate (generated from phosphorylation of glucose) is converted to ribulose-5-phosphate and CO_2_ in parallel with the production of two molecules of NADPH (Fig. 1). In the first reaction, glucose-6-phosphate dehydrogenase (G6PDH, the pacemaker enzyme of the PPP), catalyzes the oxidation of glucose-6-phosphate to 6-phosphogluconolactone with the concomitant reduction of NADP^+^. Subsequent hydrolysis of the lactone ring of 6-phosphogluconolactone is catalyzed by 6-phophogluconolactonase (or lactonase) generating 6-phophogluconolactonate, which is the substrate of the enzyme 6-phosphate gluconate dehydrogenase (6-phosphogluconic dehydrogenase, 6PGDH)^7^. This enzyme catalyzes the NADP^+^-dependent oxidation and decarboxylation of 6-phophogluconolactonate to generate NADPH, CO_2_ and ribulose 5-phosphate.

The function that the PPP plays in protection against oxidative insults has been evidenced in *Escherichia coli* (*E. coli*). Upon bacteria exposure to tellurium oxyanion (a species that generates reactive oxygen species), increased activity of the PPP, mediated by transcriptional activation of the gene encoding G6PDH, has been reported^9^. Such response was associated with a better protection of *E. coli* against tellurite-induced oxidative stress^9^. Hydrogen peroxide (H_2_O_2_) also modulates the activity of the PPP in *E. coli*^10^. Regulation of the activity of G6PDH elicited by NADPH (feedback inhibition) has been proposed as a mechanism to control the activity of the PPP under low and high levels of oxidative stress^10^.

In spite of the pivotal role of the PPP in cells, and the well documented production of reactive species in biological environments^1,8^, the effects of oxidation on G6PDH are only partially explored, and little is known about 6PGDH oxidation. We have previously investigated oxidation of G6PDH from *Leuconostoc mesenteroides* mediated by ROO^·^, singlet oxygen, and peroxynitrite^11,12^. These studies have documented a loss of enzymatic activity, with the mechanisms and patterns of oxidation depending on the oxidant, with this showing specificity of the oxidative processes for a particular protein-oxidant couple^11,12^. Such defined association (protein and oxidant) affects the biological activity, and has been observed in other systems, for example, in the oxidation of lysozyme, a well-known protein, mediated by several oxidants^13–18^.

In the present work, we hypothesized that the exposure of *E. coli* G6PDH and 6PGDH to identical concentrations of ROO^•^ would result in a differential susceptibility to enzyme inactivation. Furthermore, we postulated that the pattern of oxidation on these proteins would differ due to their different amino acid contents (14 Tyr; 12 Trp, 12 Met in G6PDH *versus* 25 Tyr; 4 Trp, and 7 Met in 6PGDH per monomer) and 3-dimensional structure. In particular, we expected that oxidation of 6PGDH, which has a high Tyr content and a lower Trp content than G6PDH, would give rise to more Tyr consumption and crosslinking via enhanced formation of di-Tyr.

## Results

### Effect of AAPH-derived ROO^•^ on the enzymatic activity of G6PDH and 6PGDH

**Figure 2:**
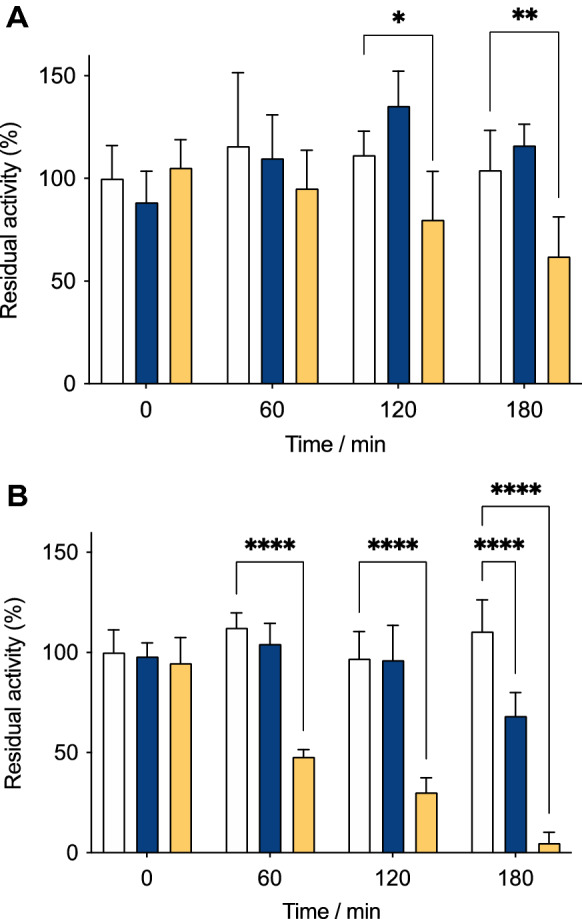
The enzymatic activities of G6PDH (panel **A**) and 6PGDH (panel **B**) are affected by AAPH-derived ROO^•^. Solutions of G6PDH (54 μM monomer) or 6PGDH (58 μM monomer) were incubated at 37ºC in phosphate buffer 75 mM, pH 7.4, in the absence (white bars) and presence of 10 (blue bars) or 100 (orange bars) mM AAPH. At defined times, aliquots were taken, the AAPH removed, and the enzymatic activity assessed by following NADPH release at 340 nm, as described in the Materials and methods. The data presented are means ± standard deviations of at least three independent experiments, each measured in triplicate. Statistical differences are indicated as follows: * p < 0.05, ** p < 0.01, and **** p < 0.0001.

The enzymatic activity of G6PDH and 6PGDH was assessed by following the release of NADPH at 340 nm in the presence of NADP^+^ and the substrates G6P and 6-phosphogluconic acid, respectively. The activity of G6PDH (expressed as residual activity, %) was not affected by incubation with 10 mM AAPH for 180 min at 37 °C (Fig. 2A), but incubation with 100 mM AAPH, over the same time frame, induced a significant loss of activity (up to 62.0 ± 15.0% of the initial value). Assuming an on–off (active–inactive) mechanism, this value implies that ~ 20 μM G6PDH were inactivated by the ROO^·^ generated from 100 mM AAPH. With 6PGDH, a higher efficiency of ROO^·^-mediated inactivation was observed, with the enzymatic activity decreased to 69.0 ± 11.0 and 4.5 ± 5.0%, after 180 min incubation with 10 and 100 mM AAPH, respectively (Fig. 2B). These values represent ~ 18 and ~ 55 μM of inactivated 6PGDH.

### SDS-PAGE studies of protein integrity

**Figure 3:**
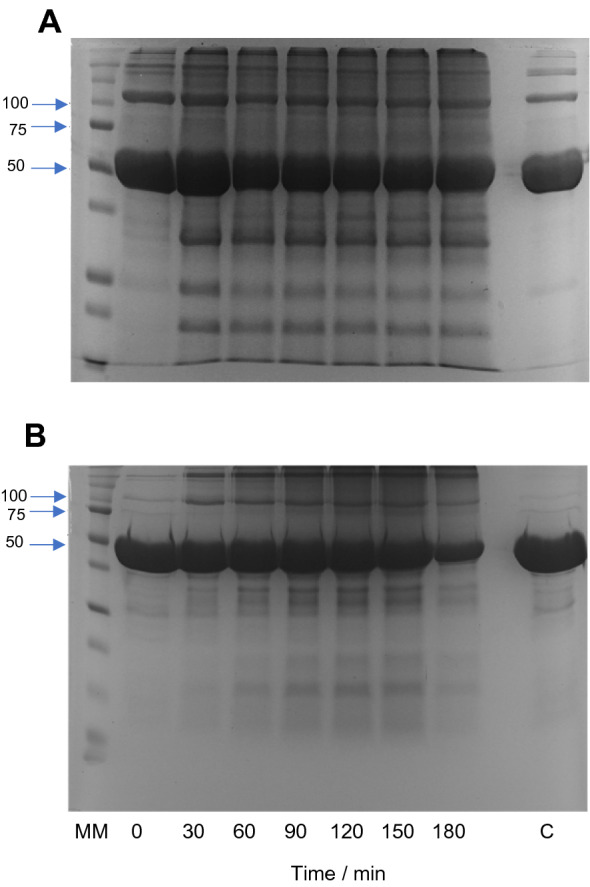
Oxidation of G6PDH and 6PGDH mediated by AAPH-derived ROO^•^ affects the molecular mass of the proteins and generates aggregates and fragments. Solutions of G6PDH (54 μM monomer) or 6PGDH (58 μM monomer) were incubated in the absence and presence of 100 mM AAPH at 37ºC in 75 mM phosphate buffer, pH 7.4. Aliquots of these solutions were taken every 30 min up to 180 min, AAPH removed as described in the Materials and methods, and samples kept at -80ºC until subjected to electrophoresis analysis. Panel A and B present SDS-PAGE of G6PDH and 6PGDH, respectively. SDS–PAGE was run under reducing conditions on 4–12% Bis–Tris acrylamide gels with 60 μg of protein loaded per well. MM: molecular mass markers, with masses indicated on left hand vertical axis. Lane C shows the electrophoretic pattern of proteins incubated for 180 min in the absence of AAPH.

Potential changes in the molecular mass of G6PDH and 6PGDH induced by ROO^·^ were examined by SDS-PAGE. Incubation of G6PDH with 10 mM AAPH (Fig. S1A) had minimal effects on the intensity of the monomeric band, but a significant decrease was observed with 100 mM AAPH (Fig. 3A). With both 10 and 100 mM AAPH, and incubation times of > 30 min, new bands corresponding to protein fragments and protein aggregates were evidenced (Fig. S1A, Fig. 3A). In the case of 6PGDH, incubation with 10 or 100 mM AAPH resulted in the detection of protein crosslinks and a low level of fragmentation (Fig. S1B, Fig. 3B). The monomeric band of 6PGDH was not significantly decreased by incubation with 10 mM AAPH, but a decrease in intensity was observed after 180 min incubation with 100 mM AAPH (Fig. 3B).

### Consumption of amino acids and quantification of oxidation products

**Figure 4:**
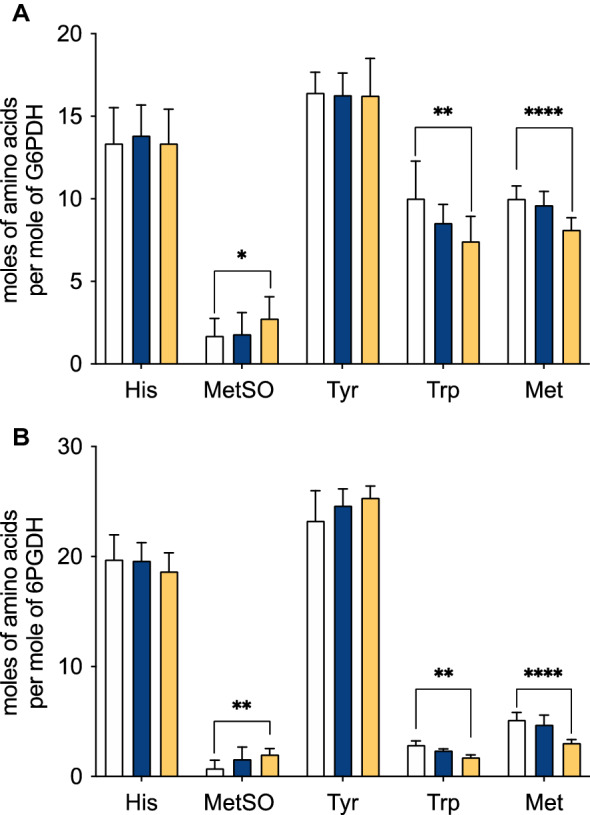
Quantification of His, Tyr, Trp, and Met modification and formation of methionine sulfoxide (MetSO) in samples of G6PDH (panel **A**) and 6PGDH (panel **B**) treated with 10 (blue) and 100 (orange) mM AAPH. G6PDH (54 μM monomer) and 6PGDH (58 μM monomer) were incubated with AAPH at 37 °C in 75 mM phosphate buffer at pH 7.4 for 180 min. Aliquots were then taken, AAPH removed, and amino acid content assessed by OPA derivatization and HPLC analysis, as described in the Material and methods. Results were normalized to the concentration of Leu residues (to compensate for any losses during processing), and are expressed as moles of residue per mole of protein. The white bars show data determined at time zero. Incubations for 180 min of proteins in the absence of AAPH showed minor changes in amino acid content (data not shown). Data correspond to means ± standard deviations of at least three independent hydrolyses, from three independent experiments. Statistical differences are indicated as follows: *p < 0.05, **p < 0.01, and ****p < 0.0001.

Amino acid consumption was quantified by OPA pre-column derivatization followed by HPLC analysis with fluorescence detection. In spite of the high Tyr content of both proteins (12 and 25 residues per monomer of G6PDH and 6PGDH, respectively), no consumption, at the analytical sensitivity of the OPA assay, was determined. In contrast, consumption of Trp, and Met was observed for both proteins (Fig. 4, Fig. S2). In the case of G6PDH, incubation with 10 and 100 mM AAPH, induced consumption of 80.0 and 140.1 μM Trp (15% and 26% of the initial Trp content), respectively (Fig. 4A, Table 1). These values represent 1.5 and 2.6 mol of Trp consumed per mole of G6PDH. In the case of Met and 10 mM AAPH, 20 μM Met was lost (17%, 0.37 mol consumed per mole G6PDH), whereas with 100 mM AAPH, 102.4 μM was lost (18.9%, 1.9 residues of Met modified per mole of G6PDH). With 6PGDH, Trp consumption of 29 μM (4.0%, 0.5 mol per mole of 6PGDH) and 63.8 μM (36.6%, 1.1 mol per mole of 6PGDH) were determined in the presence of 10 and 100 mM AAPH, respectively (Fig. 4B, Table 1). Met consumption was also detected with 6PDGH, with 22 μM (7.0%, 0.38 mol per mole of 6PGDH) and 121.8 μM (23.9%, 2.4 mol per mole of 6PGDH) determined for incubations with 10 and 100 mM AAPH after 180 min (Table 1).

**Table 1:** AAPH-derived ROO^·^ affect the amino acid content of G6PDH and 6PGDH.

AAPH (mM)	Trp		Met	
	10	100	10	100
**G6PDH**				
Consumption (μM)	80.0 (15.0%)	140.1 (26.0%)	20.0 (17.0%)	102.4 (18.9%)
Per ROO^·^	0.56	0.09	0.14	0.07
Per protein	1.5	2.6	0.37	1.9
**6PGDH**				
Consumption (μM)	29.0 (4.0%)	63.8 (36.6%)	22.0 (7.0%)	121.8 (23.9%)
Per ROO^·^	0.2	0.044	0.15	0.09
Per protein	0.5	1.1	0.38	2.4

Consumption of amino acids was assessed by OPA assay after incubation of G6PDH (54 μM) or 6PGDH (58 μM) with 10 and 100 mM AAPH for 180 min at 37 °C. Data are expressed as μM concentrations. The ratios of amino acid consumption (μM) and the total dose of ROO^·^ generated into the system (0.144 or 1.44 mM for 10 or 100 mM AAPH), and protein concentration (μM), are presented. Such ratios give values meaning the moles of amino acid consumption per mole of ROO^·^, and per mole of protein, respectively. Values in parenthesis show the % consumption of each amino acid, taking the initial values as 100%. Data are the mean of at least three independent experiments each measured in triplicate. For ease of reading only the standard deviations of loss of enzymatic activity are shown.

The formation of specific oxidation products on the side chain of Met, Tyr, and Trp of the AAPH-treated proteins were determined by LC with fluorescence or mass detection. As presented in Table 2, with 100 mM AAPH, 59.3 ± 16.2, and 69.6 ± 12.5 μM of MetSO were detected on G6PDH and 6PGDH, respectively. In contrast to these high levels of MetSO, low concentrations of the Tyr oxidation products di-Tyr, and DOPA (3,4-dihydroxyphenylalanine) were detected. The Trp oxidation product kynurenine (Kyn) was also detected with 12.7 ± 3.9 and 16.2 ± 0.5 μM determined after incubation of G6PDH and 6PGDH with 100 mM AAPH for 180 min, respectively. These levels are much lower that the extent of Trp consumption (140.1 and 63.8 μM for G6PDH and 6PGDH, respectively, Table 1), indicating that other products of Trp (e.g., hydroperoxides and alcohols^19,20^) are also generated to a significant extent. The formation of these alternative species was not investigated further, due to the absence of authentic standards, and the known instability of such hydroperoxides^19–21^.

**Table 2:** Quantification of carbonyls and specific oxidation products of Met, Tyr, and Trp in G6PDH (54 μM) or 6PGDH (58 μM) exposed to 100 mM AAPH for 180 min at 37 °C.

	MetSO	Di-Tyr	DOPA	Kyn	Carbonyls
**G6PDH**					
Production (μM)	59.3 ± 16.2	0.03 ± 0.014	< 1.0	12.7 ± 3.9	7.6 ± 0.5
Per ROO^·^	0.04	2.1 × 10^–5^	–	8.8 × 10^–3^	5.3 × 10^–3^
Per protein	1.1	5.6 × 10^–4^	–	0.24	0.14
**6PGDH**					
Production (μM)	69.6 ± 12.5	0.12 ± 0.016	< 1.0	16.2 ± 0.5	9.9 ± 2.5
Per ROO^·^	0.05	8.3 × 10^–5^	–	0.01	5.3 × 10^–3^
Per protein	1.2	2.1 × 10^–3^	–	0.28	0.17

Total carbonyls were quantified by DNPH, while MetSO was determined in hydrolysed protein samples by HPLC with fluorescence detection and OPA precolumn derivatization. Di-tyrosine (Di-Tyr), 3,4-dihydroxyphenylalanine (DOPA) and kynurenine (Kyn) were assessed by LC–MS using selected reaction monitoring (SRM) with the following transitions: 198 → 151 for DOPA; 361 → 315 for di-Tyr; 209 → 94 for Kyn. Transitions corresponding to 237 → 118 for *N*-formylkynurenine, and 407 → 203 for di-tryptophan (di-Trp) were also followed, however, no signals were detected.

**Figure 5:**
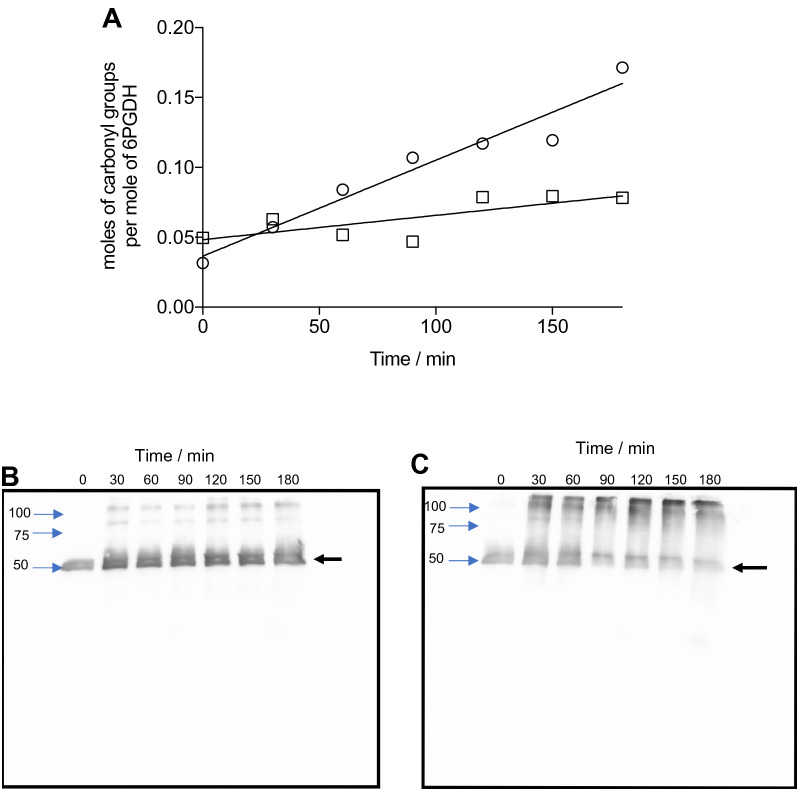
Detection and quantification of carbonyl groups on AAPH-treated 6PGDH samples. Solutions containing 6PGDH (58 μM monomer) with or without 10 or 100 mM AAPH were incubated at 37 °C in 75 mM phosphate buffer at pH 7.4. Aliquots were taken every 30 min up to 180 min, the AAPH was then removed, and samples kept at -80 ºC until analysis. Panel (**A**): quantification of the total carbonyl content on 6PGDH determined by DNPH assay incubated in the presence of 10 (open square) and 100 (open circle) mM AAPH. Panels (**B**) and (**C**): carbonyls detected by western blotting (using a commercial Oxyblot system) for samples exposed to 10 or 100 mM AAPH, respectively. On right vertical axis, black arrows indicate the band corresponding to 6PGDH monomers, while on left vertical axis, blue arrows indicate the corresponding molecular masses (obtained by comparison with molecular mass markers). For panels (**B**) and (**C**), it should be noted that images correspond to cropped and aligned WB images of membranes exposed for 50 s (as presented in Fig.S11). The data presented in panel (**A**) are the mean of at least three independent experiments, each developed in triplicate.

Carbonyl groups are generated on proteins by many types of oxidants, and are recognized markers of protein oxidation^22–24^. Consequently, the yield of such species was determined by derivatization with DNPH^24^. For both proteins, the carbonyl yields showed a linear increase on incubation with 10 or 100 mM AAPH, with similar levels determined after 180 min (Fig. 5A, Fig. S3A). With 10 mM AAPH, ~ 0.08 mol of carbonyls were detected per mole of G6PDH and 6PGDH, while with 100 mM AAPH, 0.14 and 0.17 mol of carbonyls were detected per mole of G6PDH and 6PGDH, respectively (Fig. 5A, Fig. S3A). The protein species on which these carbonyls are present was determined by using WB after separation of the protein species by SDS-PAGE. Carbonyls were detected at the position of the monomeric band of both proteins, when incubated with 10 mM AAPH for 180 min, as well as in covalent aggregates (Fig. 5B, Fig. S3B). With 100 mM AAPH, a higher accumulation of carbonyl groups was observed, with a ‘smear’ of species detected at a wide range of higher and lower molecular masses, as well as at the positions of the monomer and dimer bands of G6PDH and 6PGDH (Fig. 5C, Fig. S3C).

### Mapping of amino acid oxidation sites and protein crosslinks by LC–MS

Information on the sites of specific His, Met, Trp and Tyr modifications on G6PDH and 6PGDH induced by AAPH-derived ROO^·^ was determined by a peptide mass mapping (proteomic) approach, after digestion with LysC and trypsin^21^, with analysis performed using MaxQuant. The extents of modification at particular sites (modification occupancy) were calculated by dividing the signal intensities (area-under-the-curve) of all peptide spectral matches containing the modified position by the sum of the intensity of all peptides containing the residue under examination (i.e. both non-modified and modified). The sequence coverage obtained for control samples of G6PDH and 6PGDH was 92.5 and 99.8%, respectively, while those for the samples incubated with 10 and 100 mM AAPH were 92.5 and 92.1, and 99.8 and 98.7%, for G6PDH and 6PGDH, respectively (Fig. S4). For G6PDH, all Tyr (14) and His (6) residues were covered, whilst for Trp and Met, 11 (of 12) residues were detected. For 6PGDH all Tyr (25), His (2) and Trp (4) residues were covered, and 6 (of 7) Met residues. As presented in Figs. S5 and S6, a significant number of modified peptides were detected from the oxidized samples by LC–MS. Of particular note, is the observation that none of the Tyr, Trp or Met residues in the C-terminal regions of G6PDH and 6PGDH, were detected as modified species even with 100 mM AAPH after 180 min (Fig. S4).

**Figure 6:**
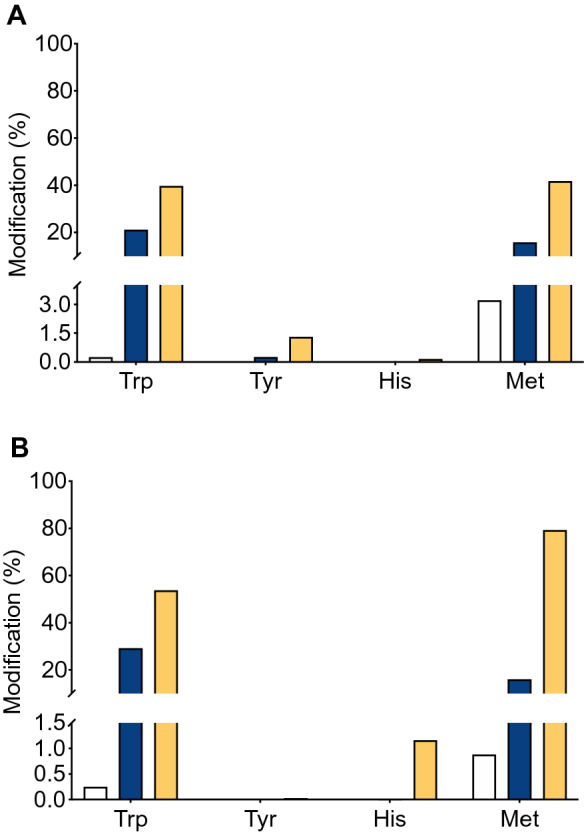
Quantification of the parent amino acids Trp, Tyr, His and Met as detected by LC–MS in G6PDH (54 μM monomer, Panel **A**) and 6PGDH (Panel **B**, 58 μM monomer) after incubation in the absence, or presence, of 10 and 100 mM AAPH at 37 °C for 180 min. The vertical axis indicates the % modification for each residue in the different conditions for control (red), 10 mM AAPH (blue) and 100 mM AAPH (orange) samples. The data are mean values obtained from the analysis of three different replicates prepared on different days. The % modification was calculated considering the full sequence of each protein G6PDH (P0AC53) and 6PGDH (P00350). The data corresponding to the quantification of the % modification of individual residues in the sequence of both proteins, with their respective standard deviations, is available in the Supplementary data.

Incubation of G6PDH with AAPH resulted in an extensive modification of Trp and Met residues as detected by LC–MS (Fig. 6A). Modification levels of 19.4% and 36.4% of the Trp residues, and 14.4% and 38.2% of Met residues, were determined after incubation with 10 and 100 mM AAPH, respectively. For Tyr and His, lower levels of modifications were detected with 1.3% and 0.2%, respectively, after incubation of G6PDH with 100 mM AAPH. Similarly, incubation of 6PGDH with 10 or 100 mM AAPH led to a significant modification of Trp and Met (Fig. 6B), with levels of 29.2% and 53.7% for Trp determined after incubation with 10 and 100 mM AAPH, respectively, and 13.7% and 67.9% for Met under the same conditions, respectively.

**Figure 7:**
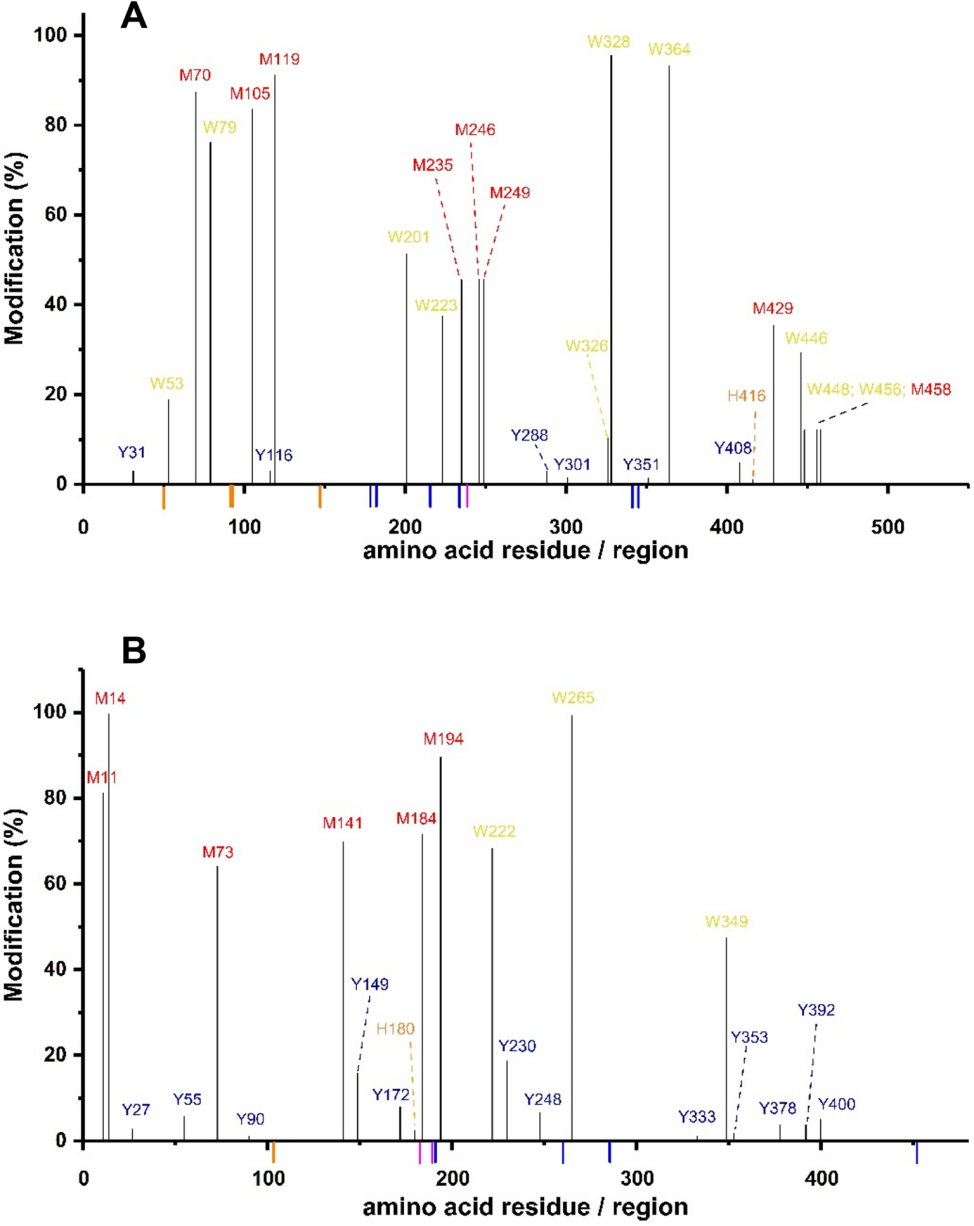
G6PDH and 6PGDH oxidation results in selective damage to amino acids within the protein sequence. G6PDH and 6PGDH (54 μM and 58 μM monomer, respectively) were incubated in the absence or presence of AAPH (10 or 100 mM, 37 °C, 180 min). Panels (**A**) and (**B**) map the oxidative changes detected at individual amino acids by LC–MS in G6PDH and 6PGDH, respectively, after incubation with 100 mM AAPH. The vertical axis indicates the % modification of the residues (Trp, Met, Tyr and His) present at specific sites, calculated as outlined in the Materials and methods from three different replicates, with the horizontal axis indicating the amino acid sequence and numbering. The positions of the binding sites for the substrate and NADP^+^ are indicated on the horizontal axis, in blue and orange, respectively. The residues that act as the proton acceptor (His239, H239) in G6PDH, and the proton acceptor (Lys183, K183) and proton donor (Glu190, E190) in 6PGDH, are indicated in pink.

Mapping of the residues modified in G6PDH (above a 10% threshold) after treatment with 100 mM AAPH (Fig. 7A, Table 3), indicated modification at the following sites: Trp53, Met70, Trp79, Met105, Met119, Trp201, Trp223, Met235, Met246, Met249, Trp326, Trp328, Trp364, Met429, Trp446, Trp448, Trp456 and Met458. The most extensive modification (> 50%) was detected at Met70, Trp79, Met105, Met119, Trp201, Trp328 and Trp364 (Fig. 7A, Fig. S5). For 6PGDH, the sites with > 10% modifications were: Met11, Met14, Met73, Met141, Tyr149, Met184, Met194, Trp222, Tyr230, Trp265, and Trp349 (Fig. 7B, Table 3, Fig. S6), with the highest extents of modification (> 50%) detected on Met11, Met14, Met73, Met141, Met184, Met194, Trp222 and Trp265.

**Table 3:** Comparison of modified amino acids at specific sites in G6PDH and 6PGDH mediated by AAPH-derived ROO^·^.

G6PDH		6PGDH	
Residue	% modification	Residue	% modification
**Methionine**		**Methionine**	
M70	High	M11	High
M105	High	M14	High
M119	High	M73	High
M235	High	M141	High
M246	High	M184	High
M249	High	M194	High
M429	High	**Trp**	
M458	High	W222	High
**Tryptophan**		W265	High
W53	High	W349	High
W79	High	**Tyrosine**	
W201	High	Y27	Low
W223	High	Y55	Low
W326	High	Y90	Low
W328	High	Y149	High
W364	High	Y172	Low
W446	High	Y230	High
W448	High	Y248	Low
W456	High	Y333	Low
**Tyrosine**		Y353	Low
Y31	Low	Y378	Low
Y116	Low	Y392	Low
Y288	Low	Y400	Low
Y301	Low	**Histidine**	
Y351	Low	H180	Low
Y408	Low		
**Histidine**			
H416	Low		

High and low refer to the % modification, using 10% as the threshold between high and low values. The numbering of the amino acids is taken from the protein sequences reported in UniProt database entry P0AC53 and P00350 for G6PDH and 6PGDH, respectively.

**Figure 8:**
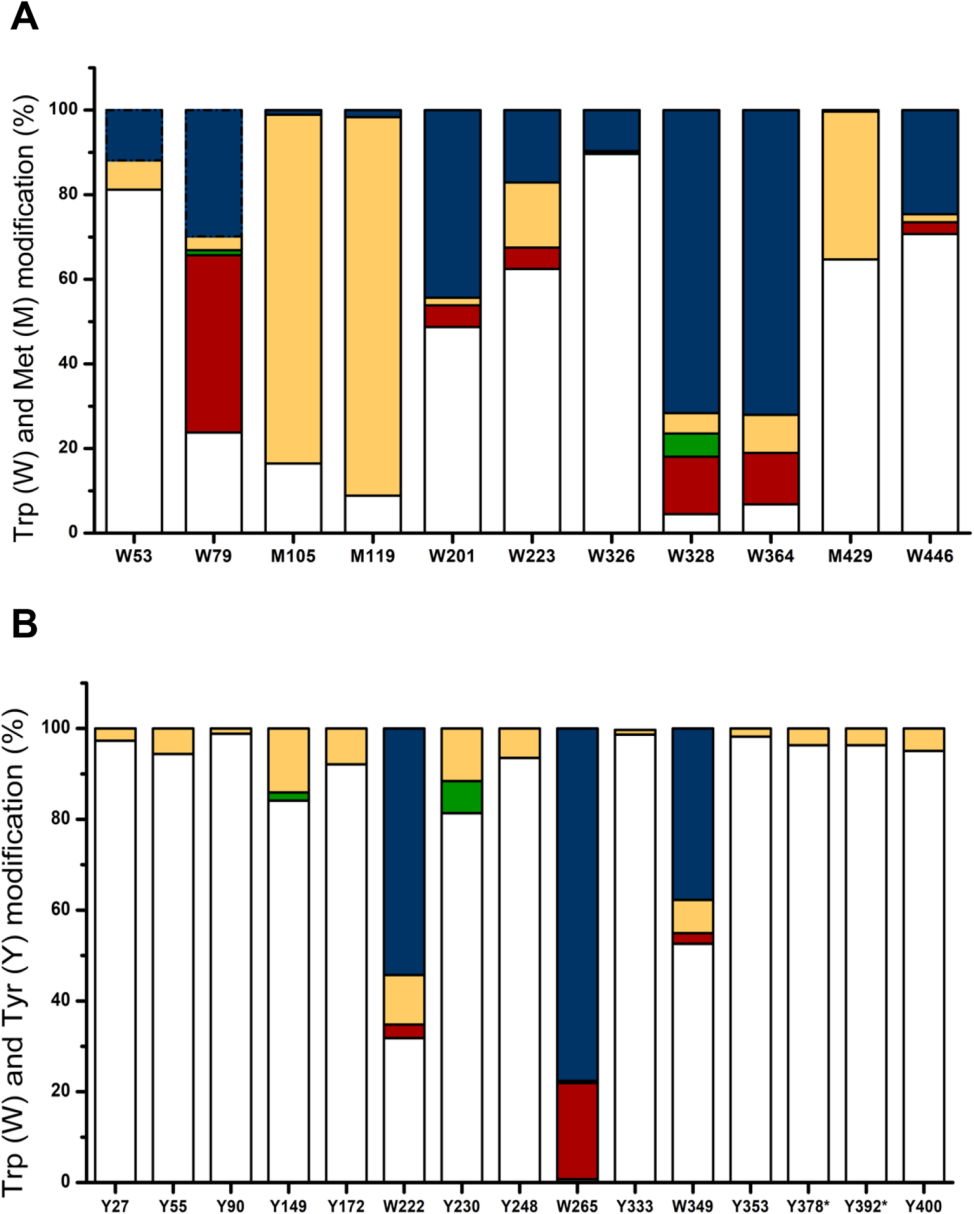
Quantification and distribution of the oxidized products generated after oxidation of Trp, Met and Tyr residues in G6PDH (Panel **A**) and 6PGDH (Panel **B**), respectively, mediated by AAPH-derived ROO^·^ (100 mM AAPH, 37 °C, 180 min). The data are presented as the average mass shifts due to the absence of specific standards to elucidate the nature of some of these species. The *m/z* + 0 data correspond to the unmodified parent Trp, Met and Tyr residues; the *m/z* + 4 (red) ions are assigned to Kyn formed on Trp oxidation; the *m/z* + 14 (accurately + 13.98, green) due to carbonyl formation; *m/z* + 16 (orange) due to the addition of a single oxygen atom (hydroxylation); *m/z* + 32 (blue) due to the addition of 2 oxygen atoms (NFK for Trp, sulfone for Met, and hydroperoxides or diols). The values correspond to mean data obtained from three different replicates carried out on different days. To facilitate data interpretation not all the mapped modifications (see Fig. 7) found in each protein are included; only those residues showing more than 1 product in G6PDH are included, whilst for 6PGDH only Trp and Tyr residues are included.

The nature and distribution of the oxidation products formed on His (*m/z* + 16 Da), Met (*m/z* + 16 and + 32), Trp (*m/z* + 4, + 14, + 16 and + 32) and Tyr (*m/z* + 14 and + 16 Da) side chains of oxidized G6PDH and 6PGDH samples were investigated by a similar approach. In AAPH-treated G6PDH, Tyr and His were detected as hydroxylated species (*m/z* + 16 Da, with DOPA being the most likely product from Tyr) (Table S1). Similarly, the predominant product generated from Met (*m/z* + 16) is assigned to the sulfoxide (MetSO, + 16 Da), though an *m/z* + 32 species (assigned to the sulfone, MetSO_2_) was detected at low levels (~ 1–2%) at Met105, Met119 and Met429 (Fig. 8A). The predominant mass shift detected for the modified Trp residues was *m/z* + 32 Da, which may be due to *N*-formylkynurenine (NFKyn), hydroperoxides and/or di-hydroxylated species (Fig. 8A). In addition, *m/z* + 4 (Kyn), *m/z* + 14 (carbonyl) and *m/z* + 16 Da shifts were also detected. Of the 10 Trp residues in G6PDH detected as modified species, only Trp79 showed a different distribution of products detected, with Kyn formation being the predominant product from this residue (~ 55.7% of the total modifications at this site) (Fig. 8A). For G6PDH, the two Trp residues with the highest levels of modification (Trp328, Trp364) showed similar levels of the *m/z* + 32 Da product (71.6% and 72.1%, respectively). Kyn also was detected at similar levels for these two residues, corresponding to 13.6% and 12.1% of the total modifications, respectively. Trp328 was also detected as a *m/z* + 14 (carbonyl) species, with this accounting for ~ 5.5% of the total product yield from this residue (Fig. 8A).

The pattern of oxidation of Trp residues in 6PGDH followed a similar trend to G6PDH, with *m/z* + 32 Da species being the most prevalent (Fig. 8B). The highest extent of modification was detected at Trp265, which was modified to *m/z* + 4 (~ 21%), *m/z* + 16 (~ 0.5%) and *m/z* + 32 (~ 78.2%) species. In contrast to G6PDH, no evidence of MetSO_2_ was detected, though extensive modification of Met to the *m/z* + 16 sulfoxide was observed. In addition, two Tyr residues (Tyr149, Tyr230) were detected as carbonyl species (*m/z* + 14, probably DOPA-quinone) along with a *m/z* + 16 species (probably DOPA) (Fig. 8B).

**Figure 9:**
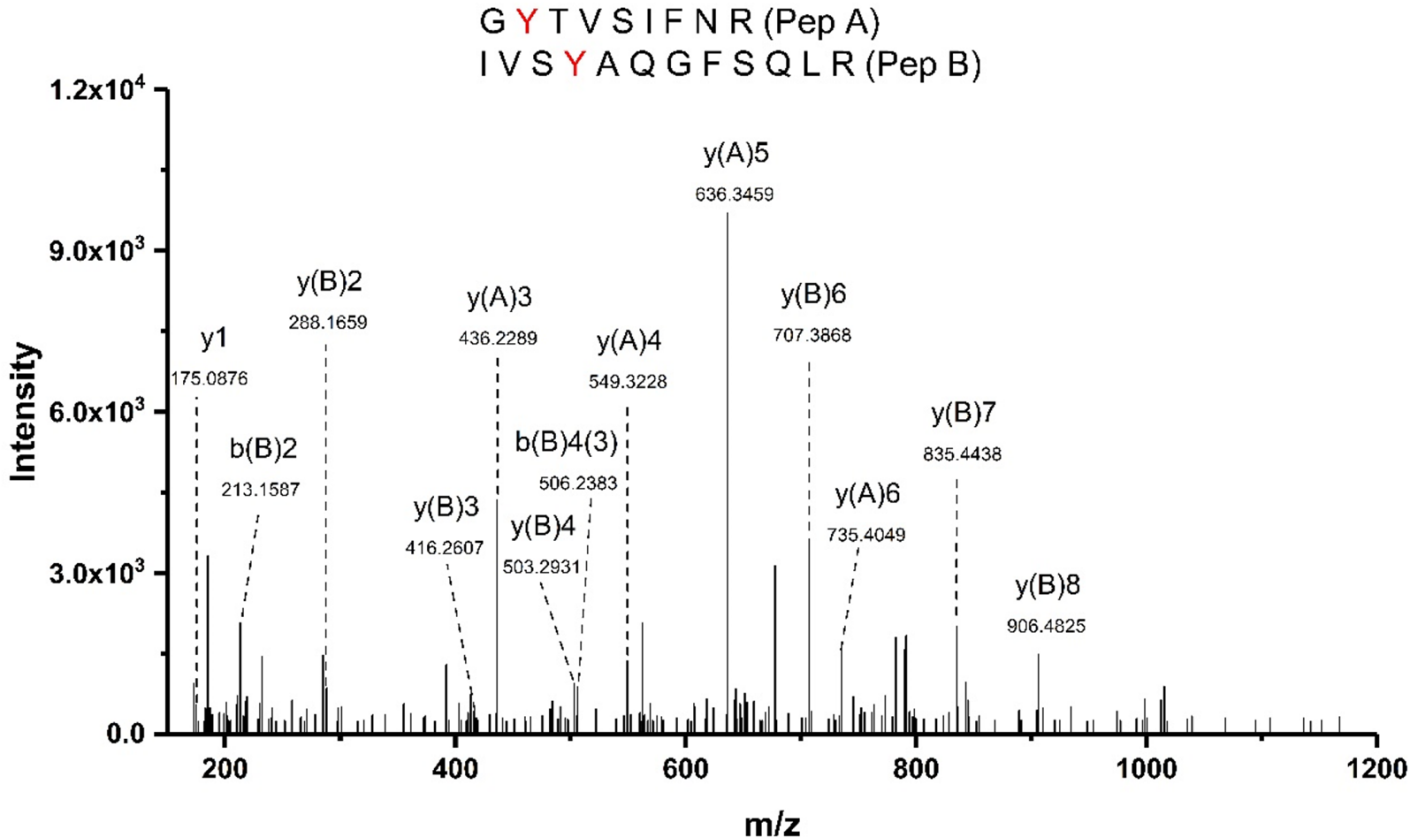
Formation of di-Tyr crosslinks partially explains the detection of aggregates formed on incubation of 6PGDH with 100 mM AAPH at 37 °C for 180 min. MS/MS spectrum of the quadruply charged crosslinked peptide (G**Y**^27^TVSIFNR) (IVS**Y**^333^AQGFSQLR) with *m/z* 606.3195. Further details are given in Table 4.

The presence of crosslinked peptides, which might rationalize the detection of dimers and higher molecular mass species by SDS-PAGE was also investigated by LC–MS (Fig. 9, Fig. S7). Searches were carried out for peptides with a mass shift corresponding to the loss of two protons (− 2.0156 Da) as this alteration arises from radical–radical recombination of two radicals (e.g. di-Tyr; di-Trp; Tyr-Trp)^25^. For both enzymes, treated with 100 mM AAPH for 180 min, formation of di-Tyr crosslinks was detected with Tyr178 and Tyr466 being involved in a covalent crosslink bond in G6PDH, and Tyr27 with Tyr333, and Tyr90 with Tyr378, in 6PGDH (Table 4). A Tyr-Trp crosslink between Trp328 and Tyr408 was also detected for oxidized G6PDH. Figure 9 illustrates the fragmentation spectrum of the crosslinked peptide (G**Y**TVSIFNR) (IVS**Y**AQGFSQLR) containing a crosslink between Tyr27 and Tyr333 in 6PGDH. The identified ions of each peptide forming the crosslink are indicated. The detected mass of this peptide of 2421.2467 Da (mass error of 1.05 ppm compared to the theoretical mass), corresponds to the expected loss of 2.01 Da compared to the two parent sequences, and is consistent with a di-Tyr crosslink. MS/MS experiments yielded a good coverage of fragment ions from each component peptide allowing the precise location of the crosslink to be determined (Fig. 9). The other crosslinked peptides detected on oxidized G6PDH and 6PGDH also showed good sequence coverage, allowing the identification of the residues involved (Fig. S7).

**Table 4:**
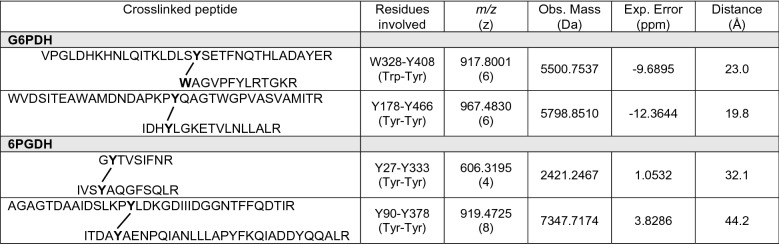
Identified crosslinked peptides in G6PDH (54 μM) or 6PGDH (58 μM) subjected to ROO^·^-mediated oxidation by 100 mM AAPH for 180 min at 37 °C.

Observed mass-to-charge ratio (*m/z*) values, with errors in parts per million, are indicated. The sequence of the crosslinked peptides (with the amino acids involved in the crosslinks highlighted in bold) are indicated. The observed mass of the crosslinks is also included. The distance from aromatic ring to aromatic ring in the crosslinked peptides was estimated from the 3D structure of G6PD (alphafold structure: P0AC53) and 6PGD (PDB: 3FWN). The crosslinks listed were found in all the replicates subjected to oxidation, and not in any of the controls.

In silico analysis of G6PDH and 6PGDH 3D structures (vide infra) allowed the determination of distances between the residues involved in these crosslinks peptides, with intramolecular distances of 32.1 and 44.2 Å determined for Tyr27 ↔ Tyr333, and Tyr90 ↔ Tyr378, respectively, in 6PGDH (Table 4). These large distances suggest that these crosslinks are intermolecular, and generated from two tyrosyl radicals on different monomers. The intramolecular distances between Trp328 ↔ Tyr408, and Tyr178 ↔ Tyr466 in G6PDH were determined as 23.0 and 19.8 Å, respectively (Table 4), implying that these crosslinks are also intermolecular in nature.

### Analysis of 3D structures of G6PDH and 6PGDH

**Figure 10:**
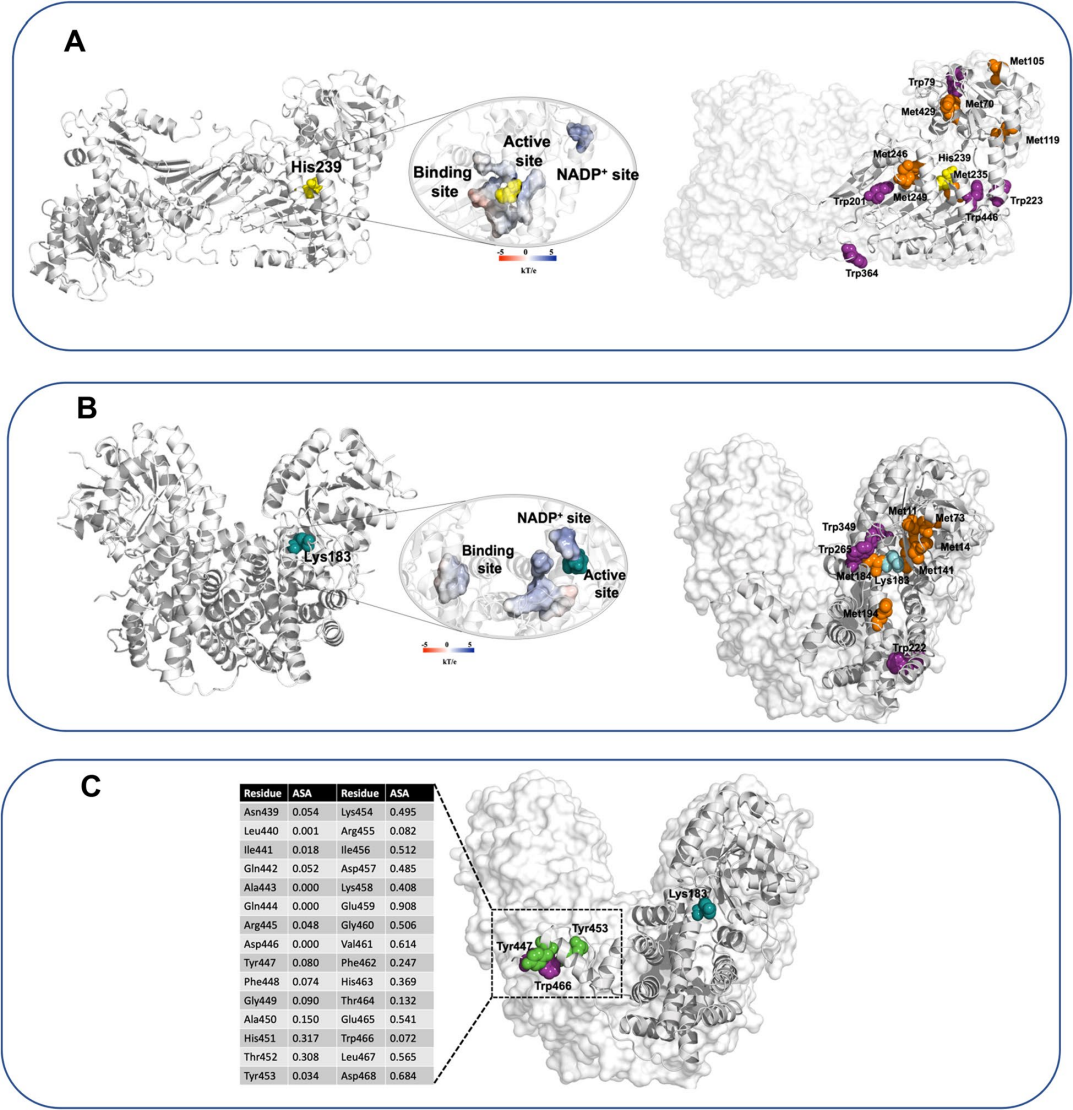
In silico analysis of the 3-D structure of G6PDH (built as described in Materials and methods section) and the crystal structure of 6PGDH (PDBid: 2ZYD). The proteins are presented as dimers, with the surface of catalytic, substrate and NADP^+^ binding sites per monomers colored based on the electrostatic potential, with a gradient from -5 kT/e (red) to 5 kT/e (blue). Amino acids involved in the catalytic mechanisms are presented; His239 for G6PDH (panel **A**, left) and Lys183 for 6PGDH (panel **B**, left). For both enzymes, residues detected as oxidized species with a modification level > 10% are presented (panels **A** and **B**, at right). Panel (**C**) shows the crystal structure of 6PGDH highlighting Tyr447, Tyr453, and Trp466, which are located at the carboxyl terminus of each monomer, which is shared with the other monomer. The table presented in panel (**C**) shows the amino acids included in this region, between Asn439 and Asp468.

Analysis of the 3D structure of dimeric *E. coli* G6PDH (built from protein monomers obtained by homology with the structure of *L. mesenteroides* G6PDH, PDB code: 1DPG, using Modeller software^26^) allowed the distances between the oxidized residues (those with > 10 and > 50% modification; Fig. 7A, Table 3) and His239, which is part of the catalytic dyad of the protein, to be estimated (Fig. S8A, Fig. 10A). These analyses (Table 5), provided distances to His239 of between 4.7 (Met235) and 37.1 (Trp364) Å. Met235, the closest amino acid to His239 (4.7 Å) is predicted to have a low exposure to the solvent, with an accessible surface area (ASA) index of 0.028. With the exception of Trp201 and Trp328, the residues which were detected with a high level of modification (> 50%), were located as considerable distances from His239 (20.6–37.1 Å), and had high solvent exposure, with ASA values of 0.138 and 0.652. These results (distance to His239 and ASA values, Table 5) suggest that modification of a single residue is not responsible for the observed inactivation of G6PDH. It is more likely that the inactivation elicited by AAPH-derived ROO^•^, arises from oxidation of multiple residues, and particularly those with high levels of modification (indicated in red in Table 5). These may also be those with a high solvent exposure.

**Table 5:** Distance between catalytic amino acids (His239 for G6PDH, Lys183 for 6PGDH), and the amino acids detected as modified species, and their respective solvent exposures, are presented.

G6PDH			6PGDH		
Residue	Distance to His239 (Å)	ASA index	Residue	Distance to Lys183 (Å)	ASA index
**Met**			**Met**		
**M70**	25.3	0.138	**M11**	19.1	0.005
**M105**	32.4	0.424	**M14**	8.8	0.240
**M119**	22.4	0.197	**M73**	9.9	0.066
M235	4.7	0.028	**M141**	6.1	0.000
M246	12.0	0.000	**M184**	10.6	0.017
M249	17.6	0.003	**M194**	11.5	0.002
M429	22.0	0.014	**Trp**		
M458	29.3	0.852	**W222**	24.8	0.024
**Trp**			**W265**	9.7	0.174
W53	30.0	0.831	W349	15.6	0.034
**W79**	32.2	0.157	**Tyr**		
**W201**	20.6	0.005	Y149	15.9	0.070
W223	20.9	0.504	Y230	12.0	0.161
**W326**	27.6	0.652			
**W328**	21.1	0.007			
**W364**	37.1	0.556			
W446	11.2	0.051			
W448	13.9	0.013			
W456	21.4	0.082			

The amino acids with modifications > 50% (see Fig. 7) believed to be involved in the loss of activity elicited by ROO^·^ are indicated in Bold text. These residues were selected on the basis of their proximity to His239 (G6PDH) or Lys183 (6PGDH) and/or their solvent exposure (determined by ASA index, 0–1). Analyses were carried out using the protein sequences reported in UniProt database entries P0AC53 and P00350, for G6PDH and 6PGDH, respectively. The sidechains of the catalytic site His239 (imidazole ring) and Lys183 (nitrogen atom) were used to calculate the distance to the Met (sulfur atom) and Trp (indole ring) residues of the oxidized residues.

In the case of *E. coli* 6PGDH, analysis of the crystal 3D structure of the dimer (Protein Data Bank, PDBid: 2ZYD, Fig. S9) showed that the amino acids which were detected with extents of modification of > 10 and > 50%, (Fig. 7B, Table 3), have distances to the active site Lys183 residue of between 6.1 and 24.8 Å, with ASA indices varying between 0.000 and 0.240 (Fig. 10B, Table 5). Interestingly, of the 11 amino acids with modification levels > 10%, 8 had modification levels > 50%, with the majority of these being Met residues (Met11, Met14, Met73, Met141, Met184, Met194, Trp22, and Trp265). Of these amino acids, Met14 is closest to Lys183 (8.8 Å), and also has a high solvent exposure (ASA index 0.240). It is therefore possible that oxidation of this residue may explain, at least in part, the inactivation of the enzyme. Nonetheless, contributions from other oxidized amino acids cannot be excluded, particularly those that are also located close to Lys183, and especially Met73, Met141 and Trp265.

Interestingly, Tyr447, Tyr453, and Trp466, which are located in the carboxyl terminus region, and which interacts closely with the adjacent monomer (Fig. 10C) were not detected as oxidized species. All of these residues showed low ASA values, implying a low solvent exposure, which is consistent with protection of these residues by the other monomer. However, it should also be noted that both Tyr453 and Trp466 are adjacent to residues with high solvent exposure, which suggests some flexibility of this region of the protein.

## Discussion

Multiple studies have investigated possible associations between G6PDH activity and oxidative stress related conditions either in eukaryotic and prokaryotic cells^7,9,10^, but the oxidation of this protein is only partially understood. Previously we have reported that oxidation of *Leuconostoc mesenteroides* G6PDH follows distinct, and alternative pathways, and subsequent consequences for protein functionality in an oxidant-dependent manner^11,12^. In order to obtain further insight into how the PPP pathway and NADPH production is altered by ROO^·^, the current study has examined the oxidation of G6PDH and 6PGDH from *E. coli*. These two proteins have a similar content of amino acids (Trp, Tyr and Met) that are likely to be susceptible to ROO^·^ oxidation, with 38 residues for G6PDH, and 36 for 6PGDH per monomer. Both proteins also contain Cys residues with G6PDH having six Cys per monomer (with only two as free Cys, the others being present in disulfides, as inferred from the 3D structure), while 6PDGH has three Cys and only one as a free residue. Free Cys residues are also a target of ROO^•^, though the present research has focused on the role of Trp, Tyr, and Met, as the observed cross-linked species are not reducible by β-mercaptoethanol and therefore unlikely to be mediated by disulfides. In spite of the similar numbers of susceptible amino acids (40 and 37 for G6PDH and 6PDGH, respectively, if free Cys are included), the relative Trp, Tyr and Met contents are different, with 14 Tyr, 12 Trp, and 12 Met in G6PDH, and 25 Tyr, 4 Trp, and 7 Met in 6PGDH. The high Tyr content in 6PGDH suggested that a high extent of oxidation might induce a high extent of protein crosslinking via di-Tyr formation from Tyr.

The results obtained on incubation of G6PDH and 6PGDH with AAPH showed clear differences in the susceptibility of these two enzymes to ROO^•^, and a higher “resistance” of G6PDH to inactivation (Fig. 2). This may be associated with the role of G6PDH as a source of NADPH in cells^7^, which could compensate for a decreased capacity of 6PGDH to generate NADPH under oxidative conditions. In fact, glycolytic cancer cells lacking 6PGDH show an accumulation of PPP intermediates, but similar NADPH production, suggesting an increased G6PDH activity^27^. Despite this, the high susceptibility of 6PGDH to ROO^•^ inactivation could be important for *E. coli*, and other type of bacteria and eukaryotic cells, for example, and particularly their capacity to synthesize nucleotides for both cellular repair and proliferation.

SDS-PAGE analyses showed that in spite of the lower susceptibility to inactivation of G6PDH, after incubation with 100 mM AAPH, protein aggregation and fragmentation was observed (Fig. 3A, Fig. S1). For 6PGDH, similar behavior was observed, with only small changes in the monomer band detected with 10 mM AAPH, but marked deceases detected with 100 mM (Fig. 3B, Fig. S1). Crosslinking of 6PDGH was detected with production of high molecular mass aggregates, however only a low extent of fragmentation was observed for 6PGDH incubated with 100 mM AAPH for 180 min (Fig. 3B). The crosslinking of both proteins may be associated with the formation of di-Tyr, di-Trp or Trp-Tyr bonds^25^, while the observed fragmentation may arise from initial oxidation at the side chains (e.g. of Trp) and subsequent transfer (propagation) of damage to the peptide backbone, as previously suggested for G6PDH^28,29^. Further information on the mechanism of inactivation, and the changes in molecular mass, was obtained by quantifying amino acid consumption^30^. In agreement with the moderate reactivity of ROO^·^^2^, only changes to Trp and Met were detected using this approach (Fig. 4, Fig. S2) with, after 180 min incubations with 100 mM AAPH, 2.6 and 1.9 mol of Trp and Met consumed per mole of G6PDH, respectively (Table 1). In contrast, 1.1 and 2.4 mol of Trp and Met were consumed per mole of 6PGDH, respectively. Two key messages arise from these data. The first is that in spite of the ability of ROO^·^ to oxidize Tyr^31,32^, and the abundance of this residue in both proteins (e.g. the 25 Tyr per monomer in 6PGDH), no consumption of this amino acid was detected. The second point is that after 180 min incubations with 100 mM AAPH, the total consumption of Trp and Met was 4.5 and 3.5 mol per mole of G6PDH and 6PGDH, respectively. Thus, there is a greater extent of damage detected on G6PDH, when compared to 6PGDH, but a greater extent of inactivation of the latter. This suggests that the damage inflicted by ROO^·^ on 6PGDH is more specific and efficient with regard to inactivation, than for G6PDH.

Specific oxidation products generated from these amino acids and others were quantified by LC–MS. In line with the lack of Tyr consumption determined by the OPA assay, only low levels of di-Tyr and DOPA were determined after incubation of both proteins with 100 mM AAPH for 180 min (Table 2). Higher amounts of the Trp-derived product Kyn were detected, however, its precursor, *N*-formylkynurenine, was not detected. This was not unexpected, as this species is readily converted to Kyn under acidic conditions^33^. Thus, the concentrations of Kyn determined are likely to represent the sum of Kyn and *N*-formylkynurenine. MetSO, the major oxidation product of Met^34^, was detected at high concentrations with 1.1 and 1.2 mol of MetSO generated per mole of G6PDH and 6PDGH, respectively, after treatment with 100 mM AAPH for 180 min (Table 2). The concentration of protein carbonyl groups, a widely employed generic biomarker of protein oxidation^35^, were also significantly elevated under these oxidation conditions, but values (0.14 and 0.17 mol of carbonyls per monomer of G6PDH, and 6PGDH, respectively) were markedly lower than the levels of MetSO (see above). Carbonyls, which showed a consistent time-dependent increase (Fig. 5A, Fig. S3), were present on both the monomer and aggregated proteins as determined by western blotting (Fig. 5B,C Fig. S3). In the case of G6PDH, incubation with 100 mM AAPH for 90 min, also resulted in the detection of carbonyl groups on protein fragments.

Consistent with the OPA assay data (Fig. 4, Table 1), the LC–MS analyses indicated significant and selective damage at Trp and Met residues, and a lower extent of modification of Tyr and His (Fig. 6). Peptide mass mapping of these modifications showed that 18 residues (10 Trp and 8 Met) on G6PDH, after incubation with 100 mM AAPH, showed > 10% modification (Fig. 7A, Table 3), with 7 showing > 50% modification (Fig. 7A and Fig. S5). For 6PGDH, 11 residues (3 Trp, 6 Met, and 2 Tyr) showed modification levels of 10%, with 8 (2 Trp and 6 Met) being modified to extents > 50% (Fig. 7B, Table 3, Fig. S6). Mapping of the sites of oxidation (Fig. 8), showed that specific Trp and Met residues in G6PDH were modified by ROO^·^ leading to Trp products with *m/z* + 4, + 14, + 16, and + 32, and MetSO (*m/z* + 16), and low levels of Met sulfone (*m/z* + 32; Fig. 8A) for two Met residues. In 6PGDH, extensive oxidation of Met to MetSO (*m/z* + 16), and at two Trp residues (Trp222 and Trp265) were detected (Figs. 7B and 8B). Interestingly, some of the Trp and Tyr residues with low levels of oxidation (with the exception of Trp328 of G6PDH) were shown to be involved in protein crosslinks. Thus, covalent crosslinks were detected for G6PDH between Trp328 and Tyr408, and Tyr178 and Tyr466, while two di-Tyr linkages (Tyr27-Tyr33 and Tyr90-Tyr378) were determined for 6PGDH (Table 4, Fig. 9, Fig. S7). The distances between these residues suggest that all of these are *intermolecular* species consistent with the SDS-PAGE data.

Analysis of the 3D structure of G6PDH showed that, with the exception of Met235, the most extensively oxidized residues are located far from the active site His239 residue (Fig. 10A), and that many of these (Met70, Met105, Met119, Trp79, Trp326 and Trp364) have a high solvent exposure (Table 5). Such exposure is likely to be of particular significance, given that the ROO^·^ employed in this study have a positive charge, and hence are likely to be primarily localized to the aqueous phase. These data suggest that, for G6PDH, ROO^·^-mediated inactivation is associated with the oxidation of multiples residues. In contrast, with 6PGDH, a more specific mechanism is proposed with oxidation of a few residues located close to the active site (Lys183, Fig. 10B), with two of these residues (Met14 and Trp265) having a high solvent exposure (Table 5). Interestingly, the low solvent exposure of the carboxyl terminus fragment in 6PGDH, which is shared with adjacent monomer, may explain why Tyr447, Tyr453 and Trp466 were unaffected by ROO^·^ (Fig. 10C). Examination of the solvent exposures of the Tyr residues showed that of the 25 residues, only 10 have ASA values in the range 0.122–0.258, while 15 have values < 0.087 (data not shown). This low exposure may explain, at least in part, why despite the high Tyr content of 6PGDH, these residues are minimally modified.

As a whole, these results contribute to our understanding of the effect that ROO^•^ have on the functionality of PPP, a pivotal metabolic pathway in nearly all cells including *E. coli*. This is of relevance to the known adaptive responses of bacteria towards oxidative stress conditions^36^. In this context, we have reported that the oxidation of specific Met residues is important in the ROO^•^-induced loss of functionality of *E. coli*^37^, and *Methanocaldococcus jannaschii* (an extremophile archea)^38^ FtsZ (filamenting temperature-sensitive mutant Z) proteins. Comparison of the data from the two FtsZ proteins showed a higher resistance to inactivation of the *E. coli* isoform. The data presented here also show a marked resistance of G6PDH (the enzyme that controls the rate of NADPH production in cells) to inactivation by ROO^·^, suggesting that *E. coli* have a capacity to resist low levels of oxidative stress.

## Conclusions

*E. coli* G6PDH and 6PGDH are affected by ROO^•^ leading to oxidation of specific amino acids and loss of enzymatic activity. In both proteins, Tyr were minimally modified, with Trp and Met being the principal targets. In the case of G6PDH, the oxidized amino acids are located distant to the catalytic site, suggesting a disperse pattern of oxidative changes, and inactivation associated with oxidation at multiple residues. In contrast, 6PGDH shows a much higher susceptibility to oxidation when compared to G6PDH, with the oxidized amino acids in 6PGDH located close to its catalytic site, with a more specific pattern of oxidation and a higher efficiency of ROO^·^-mediated inactivation. These data demonstrate that key enzymes of the PPP, which are pivotal to cell metabolism, have different susceptibilities to ROO^•^. In particular, the higher susceptibility of *E. coli* 6PGDH to damage, when compared to G6PDH, may be of relevance to understanding how cells manage and resist damage arising from widespread exposure to oxidative stress events.

## Materials and methods

### Reagents and protein

G6PDH and 6PGDH were purified from *E. coli* C41 cells as described previously^39^. After purification, the protein concentration was determined using bicinchoninic acid (BCA) (Pierce BCA protein assay, Thermo Fisher Scientific) following the manufacturer’s instructions. 2,2′-azobis(2-methylpropionamidine) dihydrochloride (AAPH), trichloroacetic acid (TCA), 6-phosphogluconic acid, glucose-6-phosphate (G6P), 2,4-dinitrophenylhydrazine (DNPH), methanesulfonic acid (MSA), tryptamine, *o*-phthaldialdehyde (OPA), methionine sulfoxide (MetSO), amino acid standards, 2-mercaptoethanol, iodoacetamide (IAM), and trifluoroacetic acid (TFA), were purchased from Sigma Aldrich. Colloidal Coomassie was purchased from BioRad.

### Oxidation of G6PDH and 6PGDH

Solutions of each protein (54 μM G6PDH, 58 μM 6PGDH as monomers), in 75 mM phosphate buffer, pH 7.4, were incubated at 37 °C for 180 min in the absence and presence of 10 or 100 mM AAPH. To prevent O_2_ depletion, the solutions were bubbled with air for 30 s every 15 min. Every 30 min, 150 μL aliquots were taken, and the AAPH removed by using a 10 kDa cut-off spin filter (Amicon, Merck) with 4 cycles of washing (with 75 mM phosphate buffer, pH 7.4) and centrifugation. The resulting samples were kept at − 80 °C until analyzed.

### Enzymatic activity of G6PDH and 6PGDH

The enzymatic activity of G6PDH and 6PGDH samples was determined by quantifying the production of NADPH via its absorbance at 340 nm^40,41^. Briefly, 450 μL of solutions (in 75 mM phosphate buffer, pH 7.4), containing 0.2 mM NADP^+^ and either 0.6 mM G6P or 6-phosphogluconic acid (for G6PDH and 6PGDH, respectively), were maintained at 25 °C in a UV cuvette in an Agilent 8453 UV–visible spectrophotometer. Background absorbance was monitored for 20 s before addition (50 μL) of diluted (500-fold) protein solutions (controls and AAPH-treated) with NADPH formation monitored at 340 nm.

### SDS-PAGE studies

Control and oxidized samples of G6PDH and 6PGDH, prepared as indicated in “SDS-PAGE studies of protein integrity” section, were analyzed by SDS-PAGE as previously reported^11,13,16,17,33^. Aliquots of protein samples (20 μL) were mixed with 7 μL of 4× loading buffer (50 mM Tris–HCl pH 6.8, 2% SDS, 10% glycerol, 1% β-mercaptoethanol, 12.5 mM EDTA, 0.02% bromophenol blue) and boiled for 5 min, then loaded onto polyacrylamide gels (4% polyacrylamide stacking gel, 12% acrylamide resolving gel). The gel was then run using a buffer containing 25 mM Tris, 400 mM Gly and 0.1% SDS, pH 8.3, at 50 mA for 1 h. Gels were stained with colloidal Coomassie following the manufacturers’ instructions.

### Analysis of amino acid consumption by HPLC with fluorescence detection and precolumn derivatization

Amino acid quantification of control and oxidized samples (prepared as indicated in “SDS-PAGE studies of protein integrity” section) was performed by HPLC with OPA precolumn derivatization as described previously^11–13^. After overnight hydrolysis using 4 M methane sulfonic acid containing 0.2% tryptamine under vacuum, the samples were neutralized with NaOH (150 μL, 4 M), filtered and diluted 20-fold with ultrapure water (Milli-Q). Aliquots (40 μL) were then transferred into HPLC vials placed in an Agilent 1260 series Infinity II multisampler set at 8 °C. Samples were analyzed using an Agilent 1200 series HPLC, coupled with a fluorescence detector (Agilent 1260). Before injection, samples were derivatized by adding 20 μL of activated *o*-phthaldialdehyde (OPA) to the HPLC vials and incubated for 1 min then injected onto a reversed phase column (Hibar® 250 × 4.3 mm RP-18 endcapped, 5 μm particle size; Phurospher® STAR; Millipore) maintained at 40 °C, eluted using a gradient buffer, and detected by fluorescence (λ_ex_ 340 nm, λ_em_ 440 nm) as described previously^11–13^. Data analysis was carried out using OpenLab Software (Santa Clara, CA). Quantification was determined from the area under the curve of the corresponding chromatographic peaks, with calibration curves constructed using commercial standards of amino acids and methionine sulfoxide.

### Determination of total carbonyl groups by spectrometric assay

The concentration of protein carbonyls was determined by derivatization with 2,4-dinitrophenylhydrazine (DNPH) and determination of the absorbance of the hydrazone products^30^. Aliquots (100 µL) of controls and oxidized G6PDH and 6PGDH (see in “SDS-PAGE studies of protein integrity” section), were mixed with 500 µL DNPH (10 mM in phosphate buffer 75 mM, pH 7.4) and incubated for 40 min. The proteins were then precipitated using 100 μL of TCA (60% w/v), and centrifuged (14,000*g*, 21 °C, 15 min). The pellets were then washed with 1 mL of cold ethanol/ethyl acetate (1:1 v/v), and re-centrifuged (14,000*g*, 4 °C, 15 min), with this process repeated twice. The pellets were then solubilized using guanidine hydrochloride (200 µL, 6 M), transferred to a 96-well plate, and the absorbance measured at 370 nm. The concentration of DNPH adducts calculated using ε 22,000 M^−1^ cm^−1 ^^30^.

### Determination of protein carbonyls by western blotting (WB)

The protein species on which carbonyls were formed was examined by WB after separation using SDS-PAGE (see “Mapping of amino acid oxidation sites and protein crosslinks by LC–MS” section), as previously reported^37,38^. Prior to loading on to the polyacrylamide gels, aliquots (30 μL) of G6PDH and 6PGDH samples (see in “SDS-PAGE studies of protein integrity” section) were mixed with 45 μL of DNPH solution (see “Determination of total carbonyl groups by spectrometric assay” section), and incubated for 15 min, at 25 °C. After electrophoresis, proteins were transferred onto Hybond®-P polyvinylidene difluoride (PVDF) membranes using a Trans-Blot Turbo Transfer System (Bio-Rad). The PVDF membrane was incubated with a polyclonal antibody to 2,4-dinitrophenylhydrazones as described in the manufacturer's instructions (Chemicon International, Temecula, CA). PVDF membranes were developed using a Pierce™ ECL Western Blotting Substrate (ThermoFisher Scientific) as described by the manufacturer. Images presented in Fig. 5 and Fig. S3 were taken with 50 s exposure. Colors of these images were inverted color, and images chopped and aligned as depicted in Fig. S10. Figure S11 shows WB images taken by employing different exposure of membranes (50, 120 and 180 s), with those obtained after 50 s showing the lowest contrast.

### Mapping of modified residues and quantification of oxidation products by LC–MS

Side-chain oxidation products on G6PDH and 6PGDH were examined by LC–MS analysis of peptides released by enzymatic digestion (LysC and trypsin) using a paramagnetic bead–based approach (SP3)^21^. Briefly, control and oxidized protein samples (25 μg) were subjected to reduction using DTT (45 mM, 45 min, 21 °C), and subsequent incubation with iodoacetamide (IAM, 90 mM, 60 min, 21 °C in the dark) in in 50 mM Tris buffer containing 8 M urea, pH 8.0. SP3 solution (2 μL of a 50 μg μL^−1^ stock) was then added, followed by 50 μL ethanol. This solution was incubated for 5 min in a thermomixer at 20 °C and 1000 rpm. The tubes where then placed in magnetic racks and incubated until migration of the magnetic beads to the wall of the tubes was complete. The supernatant was discarded, and the beads washed twice with 180 μL of 80% v/v ethanol/water. After the second wash, 0.25 μg of LysC (25 μL, in 50 mM Tris–HCl containing 6 M urea, pH 7.5) were added and incubated for 3 h at 37 °C, before addition of 125 μL of 50 mM Tris–HCl (pH 7.5) solution containing 0.5 μg trypsin. The samples were then incubated overnight at 37 °C, and subsequently centrifuged (20,000*g*, 1 min). The supernatants, containing the released peptides, were then transferred to fresh tubes, acidified with trifluoroacetic acid (TFA), and subjected to StageTip solid-phase extraction on activated Empore C18 reversed-phase discs (3 M, St. Paul, MN, USA), with the samples eluted using 50 μL of 0.5% v/v TFA in 80% v/v acetonitrile. The eluents were dried down (Speedvac™ concentrator, 10 min) and samples re-suspended in 50 μL of 0.1% v/v formic acid in H_2_O. Samples were analyzed on an Impact II ESI-QTOF mass spectrometer (Bruker Daltonics, Bremen, Germany) in the positive ion mode with a Captivespray ion source connected on-line to a Dionex Ultimate 3000 chromatography system (Thermo Fisher Scientific). Analytes were separated using a Luna column (Phenomenex) at 20 °C with a flow rate of 20 μL min^−1^ by gradient elution using 0.1% formic acid (Solvent A) and 80% acetonitrile/0.1% formic acid (Solvent B).

Data analysis was performed using MaxQuant (version 1.6.1.0) with semi-specific tryptic constraints, a 1% peptide level false discovery rate and a maximum of 3 missed cleavages, 5 modifications per peptide and max peptide mass of 10,000 Da. Carbamidomethylation of Cys was used as a fixed modification for all samples. The following variable modifications were examined: Met, *m/z* + 16 (sulfoxide) and *m/z* + 32 (sulfone); Trp, *m/z* + 4 (kynurenine, Kyn), *m/z* + 13.98 (carbonyl formation), *m/z* + 16 (addition of a single oxygen atom, hydroxylation) and *m/z* + 32 (addition of 2 oxygen atoms, *N*-formylkynurenine and dihydroxylation); Tyr, *m/z* + 16 (addition of a single oxygen atom, to give the hydroxylated product DOPA), and *m/z* + 13.98 (carbonyl formation); His, *m/z* + 16 (addition of a single oxygen atom, hydroxylation)^11,13^.

### Analysis of protein crosslinks by LC–MS

Mass spectrometric analysis of crosslinked peptides was performed on released peptides (see “Mapping of modified residues and quantification of oxidation products by LC-MS” section) as reported previously^12^. MS and MS/MS data were subjected to database searches using the software MassAI (Univ. of Southern Denmark, April 2017) with the following settings: fixed (carbamidomethylation of Cys) and variable (Met oxidation: + 16 or + 32 Da, His oxidation: + 16 Da; Tyr oxidation: + 14 and + 16 Da; Trp oxidation: + 4, + 14, + 16 and + 32 Da) modifications. A maximum of 2 missed enzymatic cleavages; parent mass tolerance of 20 ppm; MS/MS peak tolerance 0.05 m*/z*. Dityrosine (di-Tyr), tryptophan–tryptophan (di-Trp), tyrosine–tryptophan (Tyr–Trp), tyrosine–lysine (Tyr–Lys), tryptophan–lysine (Trp–Lys), histidine-histidine (His-His) and histidine–lysine (His–Lys) were selected as potential crosslinks.

### Analysis of three-dimensional (3D) structures of G6PDH and 6PGDH

Rendering of the 3D structure of *E. coli* G6PDH was performed by homology modeling using Modeller software (v9 19x)^26^. For this, the sequence of the G6PDH protein from *E. coli* was aligned with the sequence of the crystal A chain of the G6PDH protein from *L. mesenteroides* (PDB code: 1DPG), which has a 31% sequence identity. Using Modeller, a total of 1000 models were made and the structure with the lowest zDOPE was chosen. The energy of the structure was minimized by solvating the protein in a box of water at 25 °C using 10,000 minimization steps in NAMD2. The protein dimer was made by aligning 2 strands copied from the *E. coli* G6PDH protein with the crystal structure of G6PDH from *L. mesenteroides*. The resulting *E. coli* G6PDH monomer structure obtained by this approach was identical to that from Alphafold (Uniprot code: P0AC53, https://alphafold.ebi.ac.uk/). The 3D description of *E. coli* 6PGDH was obtained from the Protein Data Bank (PDBid: 2ZYD) at 1.5 Å of resolution. Water molecules and phosphate groups located inside each monomer were removed. G6PDH and 6PGDH dimers were analyzed using a H++ server in order to add missing hydrogen atoms according to the experimental pH^42^. In order to examine the charge distribution in the binding cavity, studies of the electrostatic potential were carried out using Adaptive Poisson–Boltzmann Solver (APBS)^43^. Accessible Surface Area (ASA) values were calculated using the free server http://cib.cf.ocha.ac.jp/bitool/ASA. All figures were rendered using the Pymol program (Pymol Molecular Graphics System, Version 2.0 Schrödinger, LLC).

### Statistics

All data were processed using GraphPad software version 8.0, and are expressed as the mean of at least three independent experiments each measured in triplicate. Statistical analysis was carried using a one-way ANOVA test with Holm–Šídák’s post-hoc test, with *p* < 0.05 taken as statistically significant.

## Supplementary Information

Supplementary Information.

## Data Availability

The datasets generated during and/or analyzed during the current study are available from the corresponding author on reasonable request.
