# Supplementary material for: Implications of differential peroxyl radical-induced inactivation of glucose 6-phosphate dehydrogenase and 6-phosphogluconate dehydrogenase for the pentose phosphate pathway
Source: Sci Rep. 2022 Dec 7;12:21191. doi: 10.1038/s41598-022-25474-x (PMC9729611; doi:10.1038/s41598-022-25474-x)
Supplement: Supplementary file 1 — Supplementary Information. [file 41598_2022_25474_MOESM1_ESM.docx]

Implications of differential peroxyl radical-induced inactivation of glucose 6-phosphate dehydrogenase and 6-phosphogluconate dehydrogenase for the pentose phosphate pathway

Supplementary information

Juan Sebastián Reyes,^a^ Eduardo Fuentes-Lemus,^b^, Juan David Figueroa,^a^ Javier Rojas,^a,c^ Angélica Fierro,^c^ Felipe Arenas,^d^ Per Hägglund,^b^ Michael J. Davies,^b^ Camilo López-Alarcón^a*^

Author to whom correspondence should be addressed: C. López-Alarcón; e-mail: [clopezr@uc.cl](mailto:clopezr@uc.cl)


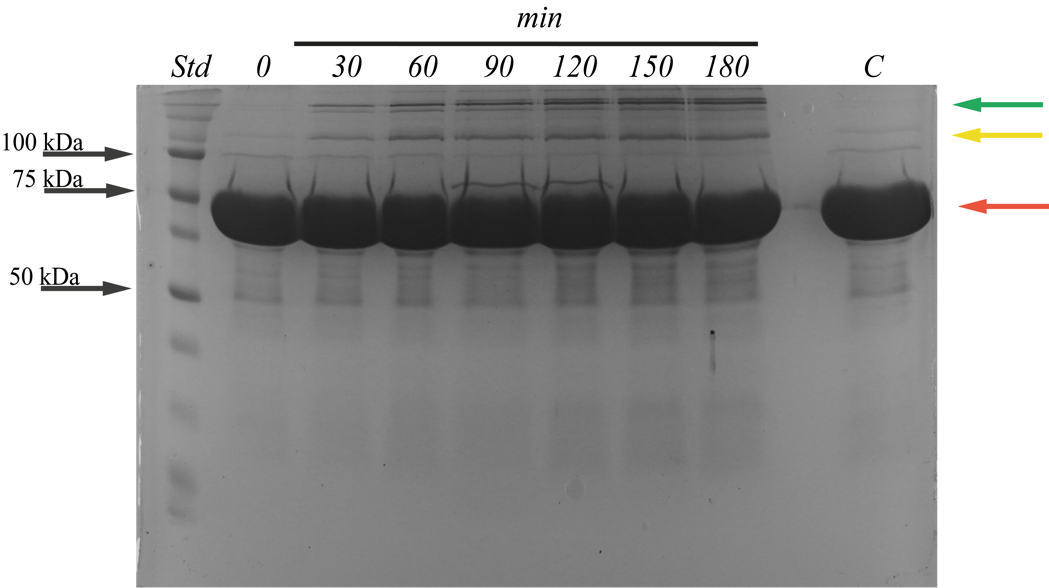

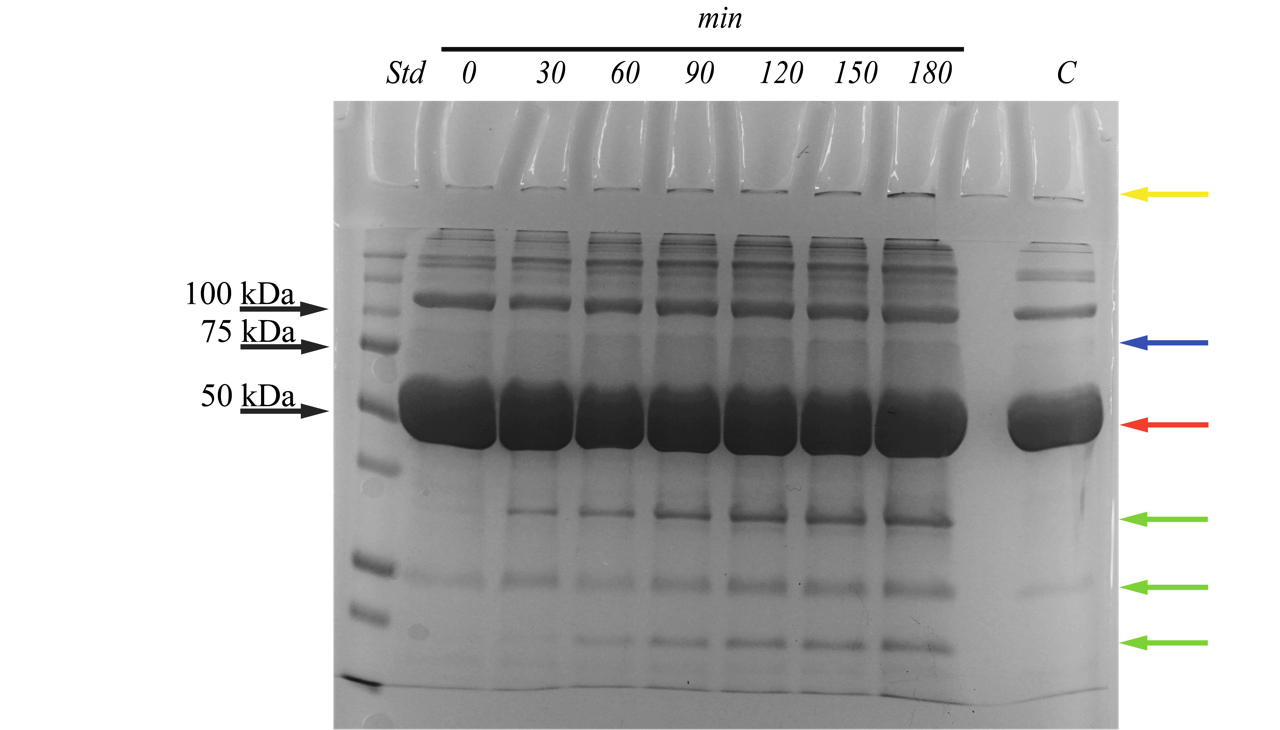


**A**

**B**

50

75

100

50

75

100

MM 0 30 60 90 120 150 180 C

Time / min

**Figure S1:** SDS-PAGE gels of G6PDH (54 μM monomer, panel A) or 6PGDH (58 μM monomer, panel B) incubated at 37 ºC in 75 mM phosphate buffer (pH 7.4) in the absence and presence of 10 mM AAPH. SDS–PAGE was carried out as described in the Materials and methods. MM: molecular mass markers, with particular masses (KDa) indicated at the left hand of vertical axis. Lane C shows the electrophoretic pattern of proteins incubated for 180 min in the absence of AAPH (control solutions).

**A**

**B**

**Figure S2:** Extent of amino acids modification and formation of methionine sulfoxide (MetSO) in samples of G6PDH (panel A) and 6PGDH (panel B) treated with 10 (blue) and 100 (orange) mM AAPH. G6PDH (54 μM monomer) and 6PGDH (58 μM monomer) were incubated with AAPH at 37 °C in 75 mM phosphate buffer at pH 7.4. After 180 min, aliquots were taken, the AAPH was removed, and amino acid content assessed by OPA derivatization and HPLC analysis, as described in the Materials and methods. Results were normalized to the concentration of Leu residues, and are expressed as moles of residues per mole of protein. White bars show data determined at t = 0 min. Incubations of the proteins for 180 min in the absence of AAPH showed minor changes in the amino acid content (data not shown). Data correspond to means ± standard deviations of at least three independent hydrolyses from three different experiments.

**A**


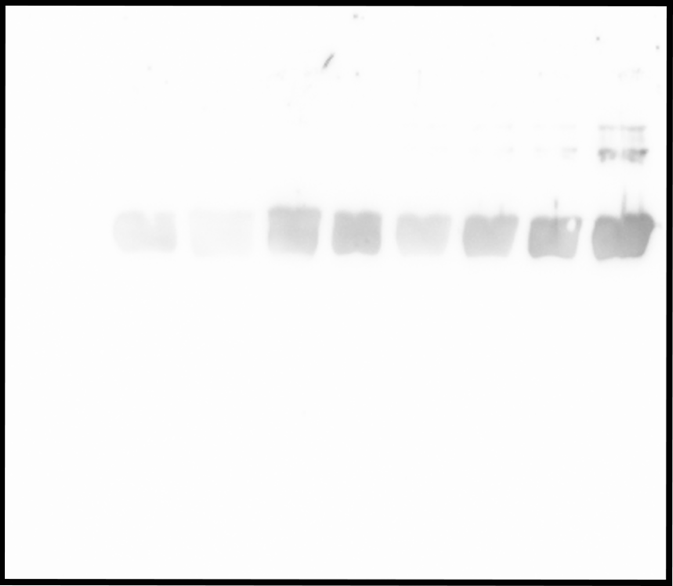


**B**

Time / min

C 0 30 60 90 120 150 180

50

75

100

**C**


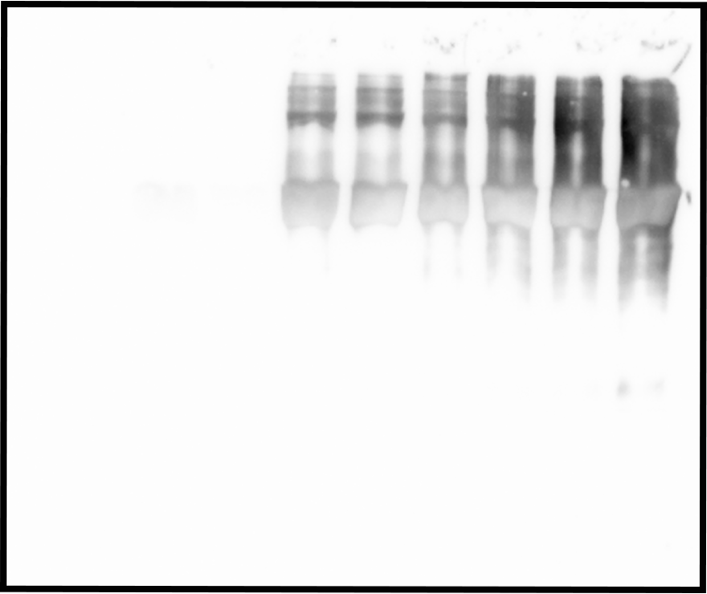


50

75

100

Time / min

C 0 30 60 90 120 150 180

**Figure S3:** Detection and quantification of carbonyls on AAPH-treated G6PDH. Solutions containing G6PDH (54 μM monomer) with or without AAPH were incubated at 37 °C in 75 mM phosphate buffer at pH 7.4. Aliquots were taken at the indicated times, the AAPH was removed, and samples kept at -80ºC until analysis. Panel A: quantification of the total content of carbonyl groups by DNPH assay for G6PDH incubated in the presence of 10 (🞎) and 100 (⭘) mM AAPH. Panels B and C: carbonyl groups detected by western blotting (using a commercial Oxyblot system, with images obtained after 50 s exposure of membranes) for samples exposed to 10 or 100 mM AAPH, respectively. Arrows indicate the masses corresponding to G6PDH monomers. For panels B and C, it should be noted that the contrast of the images is automatically adjusted by the analysis software and hence the two panels cannot be easily compared. Data presented in Panel A are the mean of at least three independent experiments each developed in triplicate.

MAVTQTAQACDLVIFGAKGDLARRKLLPSL**Y**QLEKAGQLNPDTRIIGVGRAD**W**DKAAYTKVVREALETFMKETIDEGL**W**DTLSARLDFCNLDVNDTAAFSRLGAMLDQKNRITIN**Y**FAMPPSTFGAICKGLGEAKLNAKPARVVMEKPLGTSLATSQEINDQVGE**Y**FEECQV**Y**RIDHYLGKETVLNLLALRFANSLFVNNWDNRTIDHVEITVAEEVGIEGR**W**GYFDKAGQMRD**M**IQN**H**LLQILCMIAMSPPSDLSADSIRDEKVKVLKSLRRIDRSNVREKTVRGQ**Y**TAGFAQGKKVPG**Y**LEEEGANKSSNTETFVAIRVDIDNWR**W**AGVPFYLRTGKRLPTKCSEVVV**Y**FKTPELNLFKESWQDLPQNKLTIRLQPDEGVDIQVLNKVPGLDHKHNLQITKLDLS**Y**SETFNQT**H**LADAYERLLLETMRGIQALFVRRDEVEEAWK**W**VDSITEA**W**A**M**DNDAPKPYQAGTWGPVASVAMITRDGRSWNEFE

**A**

**B**

MSKQQIGVVGMAVMGRNLALNIESRG**Y**TVSIFNRSREKTEEVIAENPGKKLVPY**Y**TVKEFVESLETPRRILLMVKAGAGTDAAIDSLKP**Y**LDKGDIIIDGGNTFFQDTIRRNRELSAEGFNFIGTGVSGGEEGALKGPSIMPGGQKEAYELVAPILTKIAAVAEDGEPCVT**Y**IGADGAG**H**YVKMV**H**NGIEYGDMQLIAEAYSLLKGGLNLTNEELAQTFTEWNNGELSS**Y**LIDITKDIFTKKDEDGN**Y**LVDVILDEAANKGTGK**W**TSQSALDLGEPLSLITESVFARYISSLKDQRVAASKVLSGPQAQPAGDKAEFIEKVRRALYLGKIVS**Y**AQGFSQLRAASEEYNWDLN**Y**GEIAKIFRAGCIIRAQFLQKITDA**Y**AENPQIANLLLAP**Y**FKQIADD**Y**QQALRDVVAYAVQNGIPVPTFSAAVAYYDSYRAAVLPANLIQAQRDYFGAHTYKRIDKEGVFHTEWLD

**Figure S4:** Sequence coverage of G6PDH (Panel A, P0AC53) and 6PGDH (Panel B, P00350) detected by LC-MS analysis. The amino acids and regions not covered are indicated in light grey. In bold orange: residues found oxidized only in the 100 mM AAPH condition; in bold and red text: residues found oxidized in 10 and 100 mM AAPH conditions; in blue: residues found oxidized in all the samples, including control samples. In green: Trp (W), Tyr (Y), His (H) and Met (M) residues that were found unmodified in all the samples.


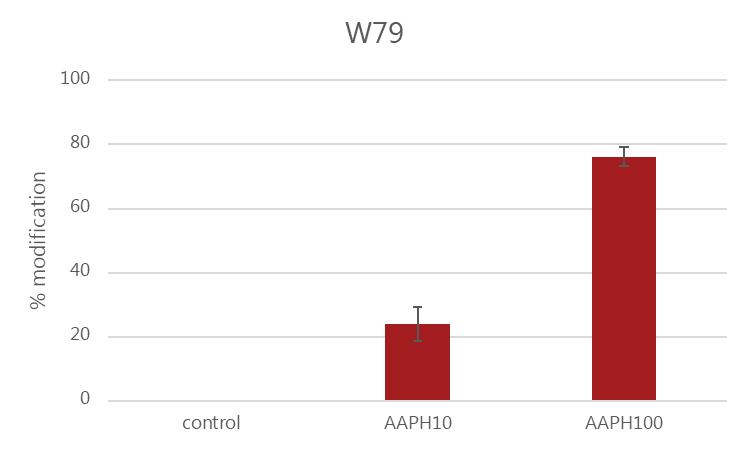

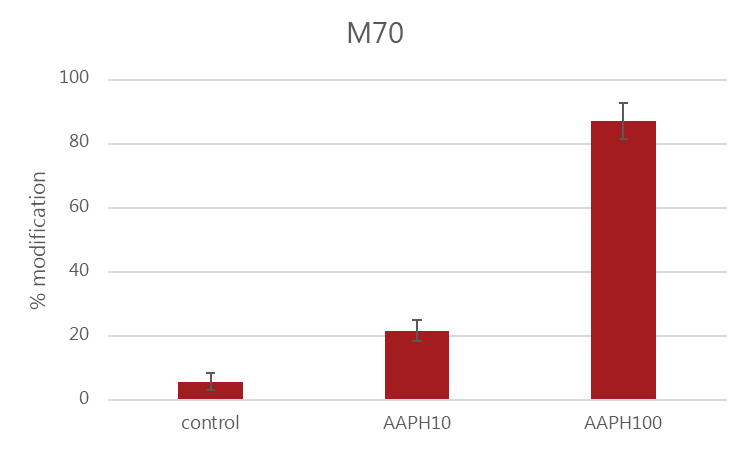

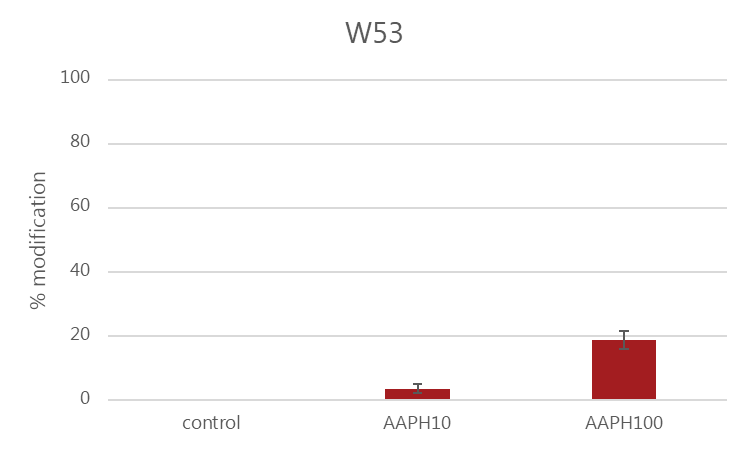

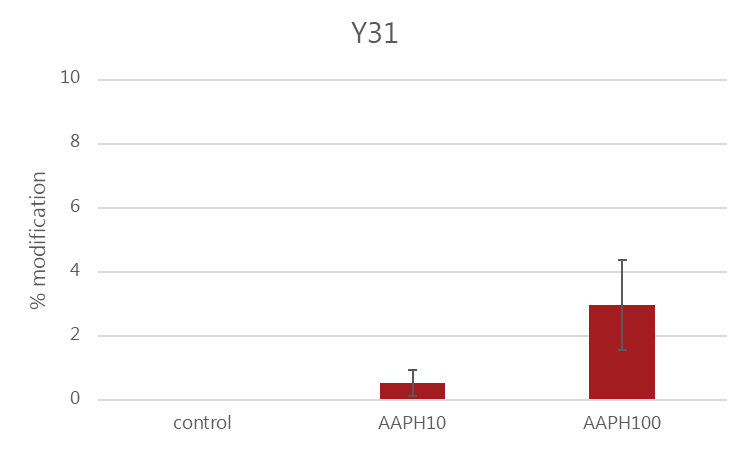


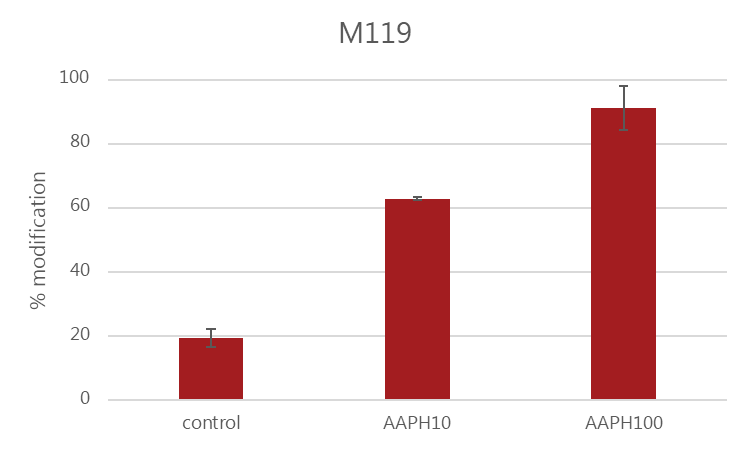

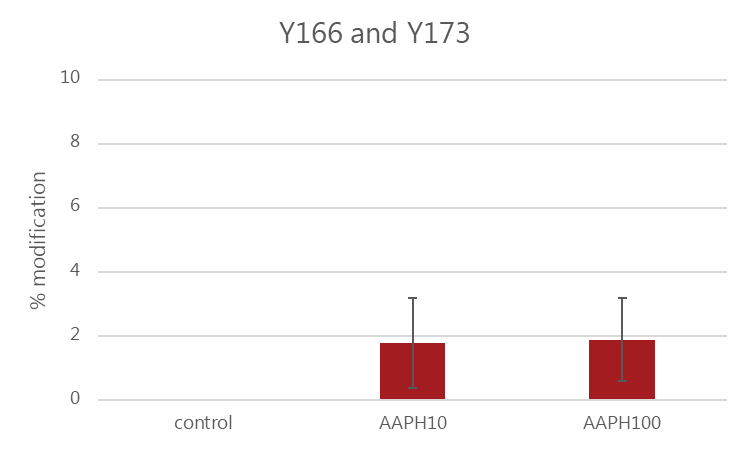

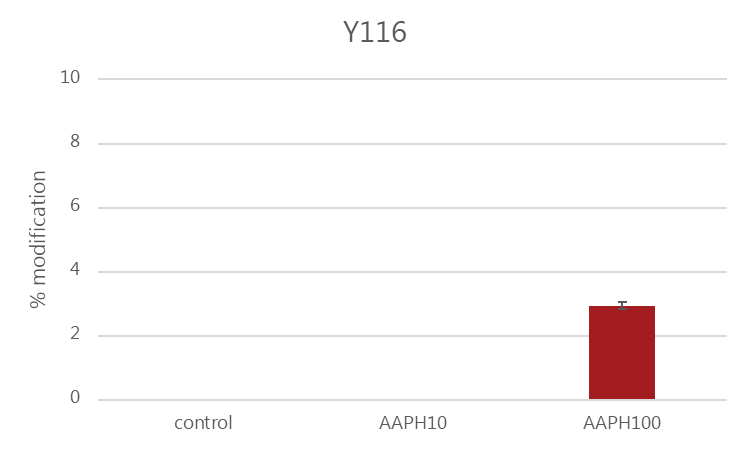

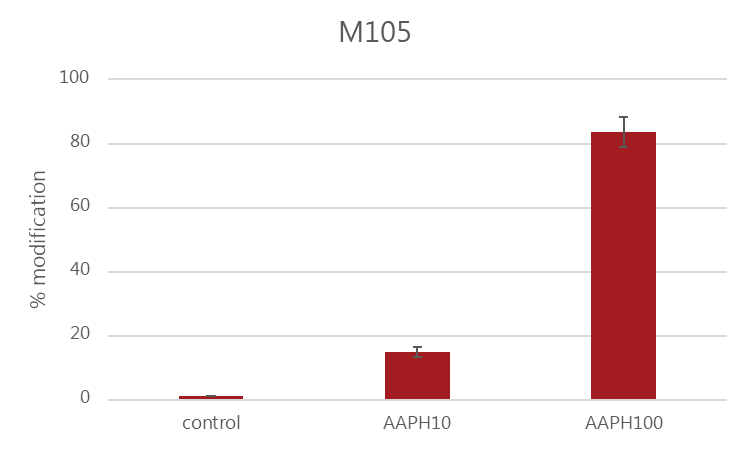


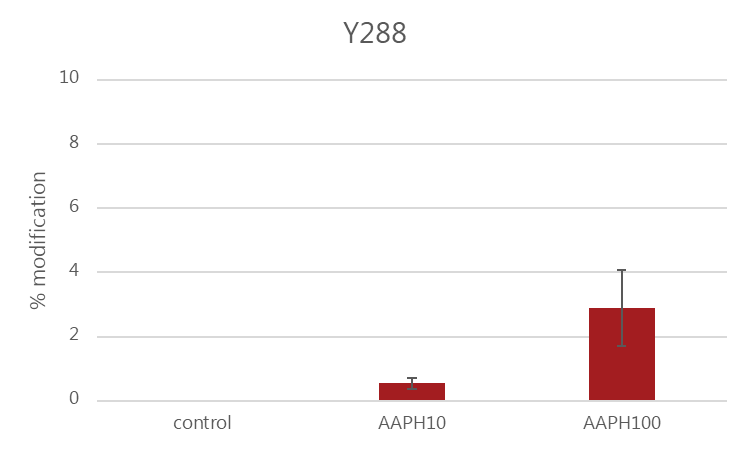

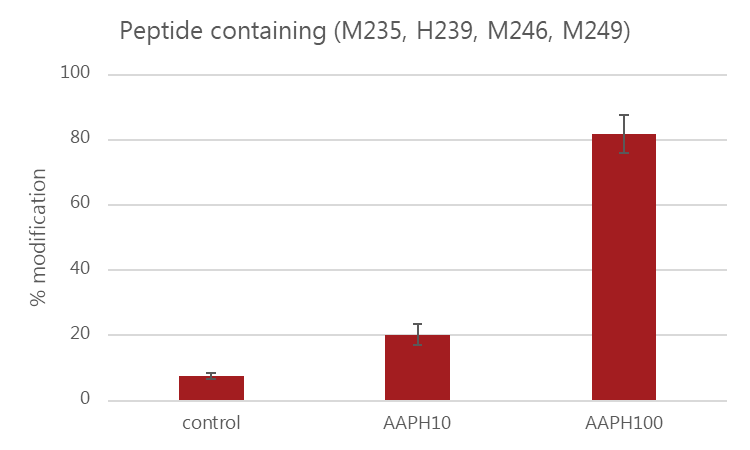

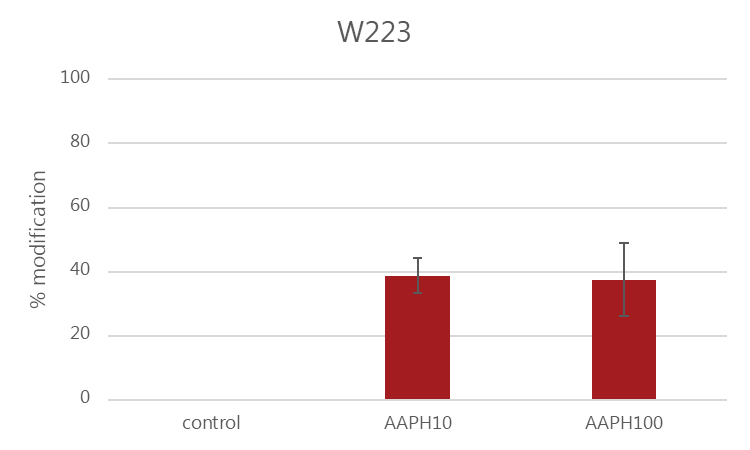

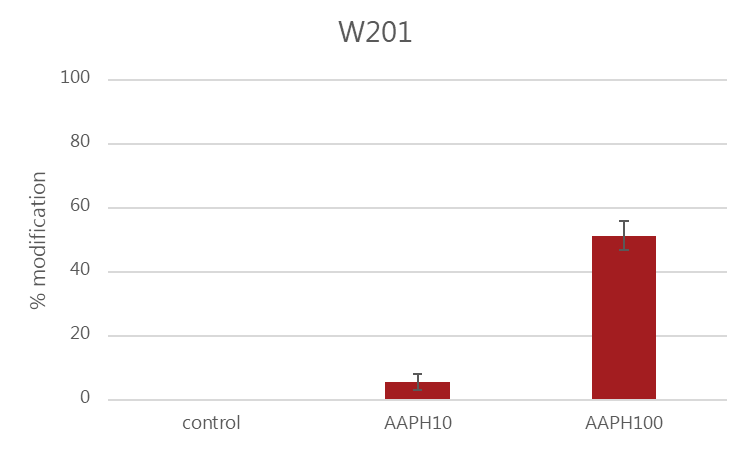


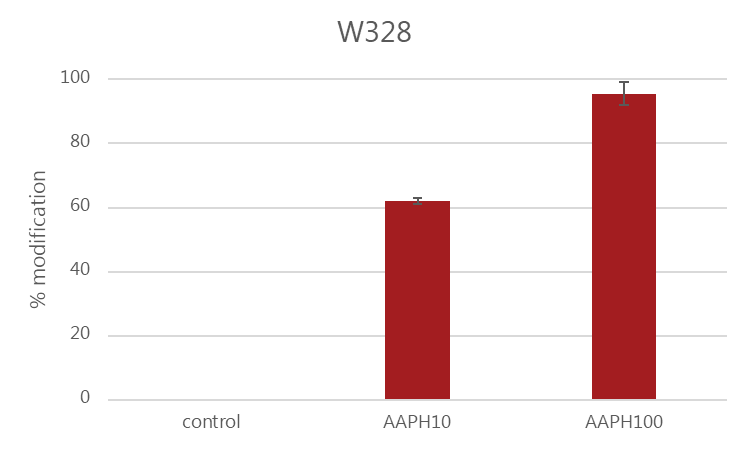

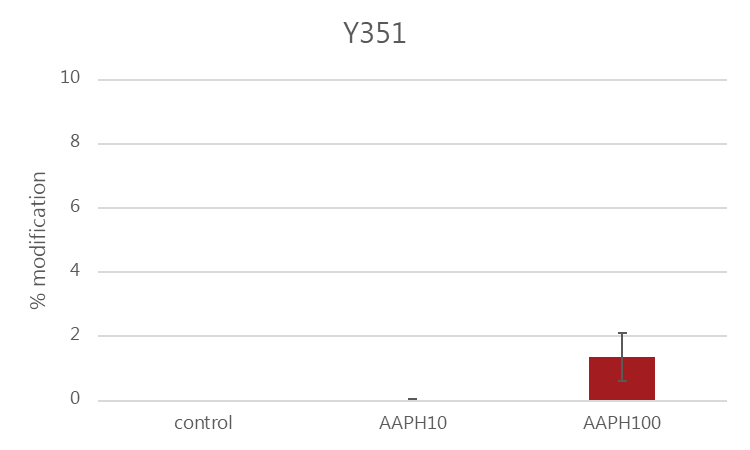

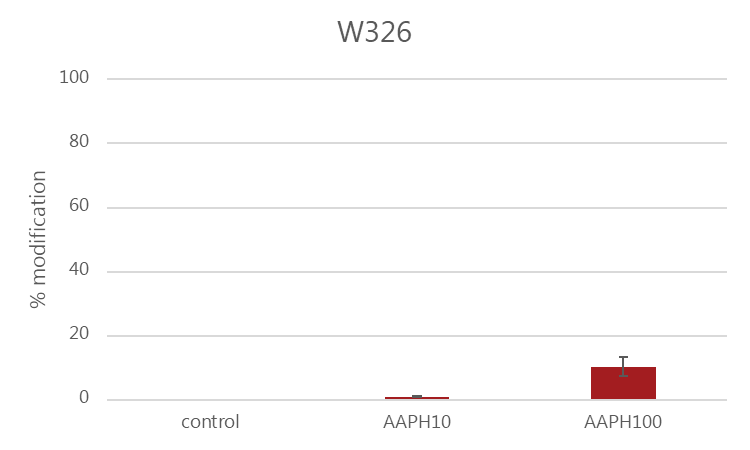

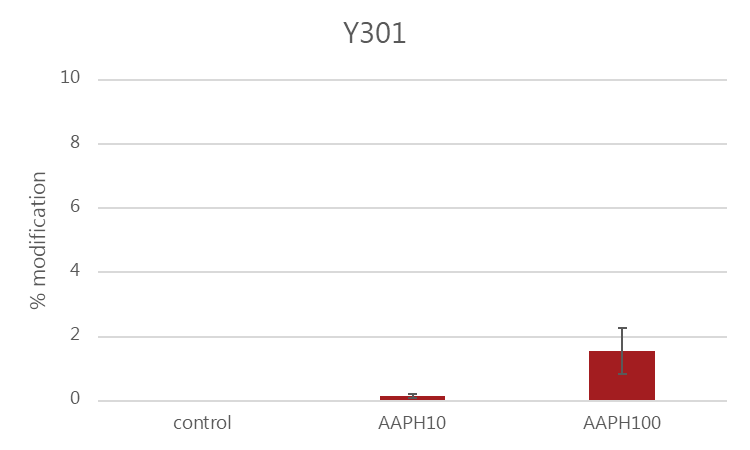


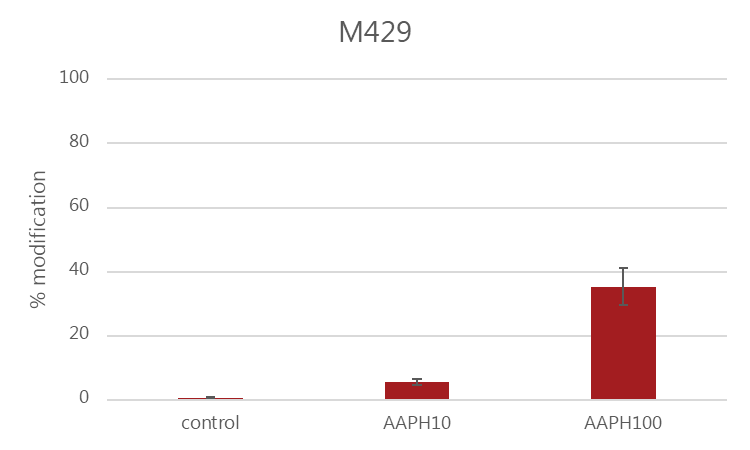

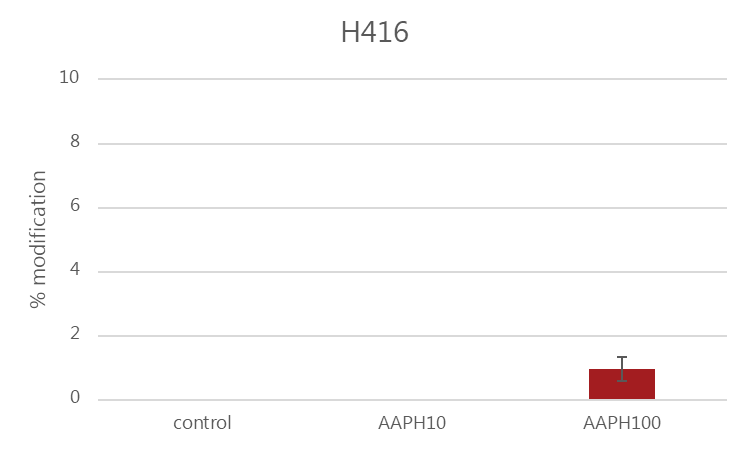

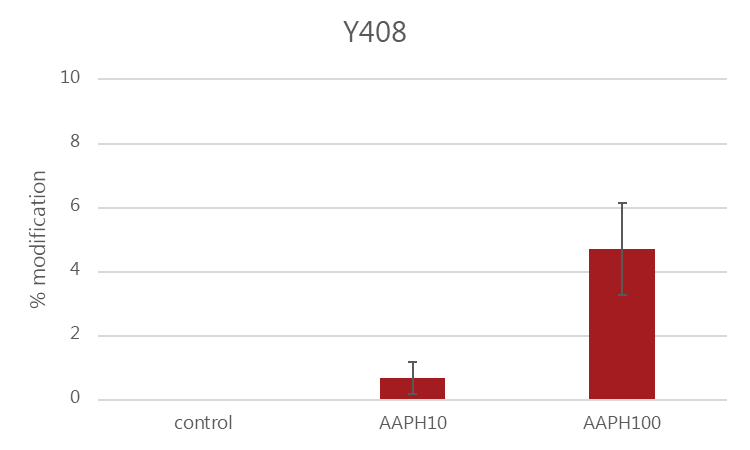

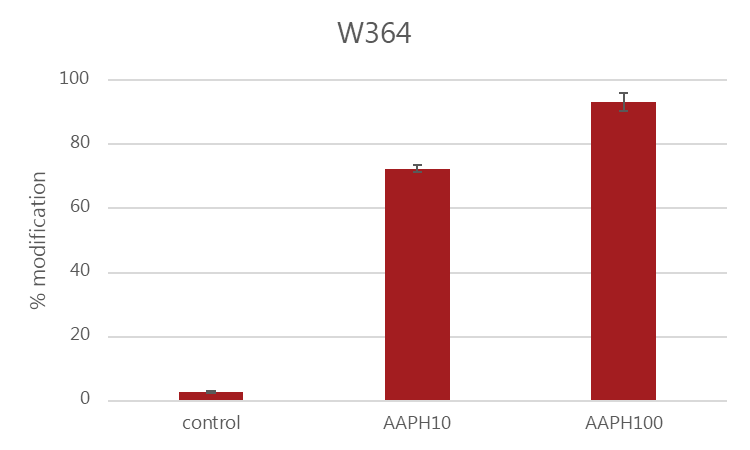


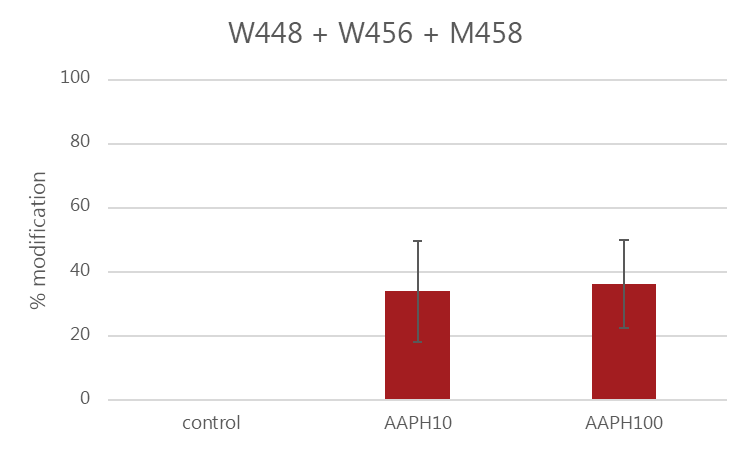

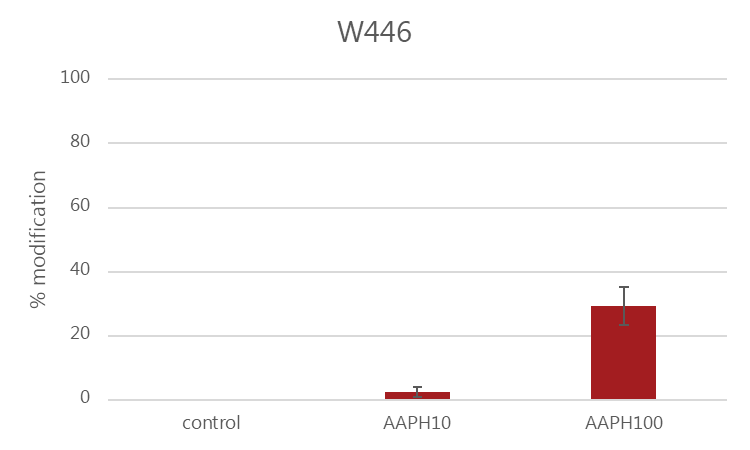


**Figure S5:** Quantification of the % modification of the peptides containing Trp, Met, Tyr and/or His residues obtained after digestion of G6PDH. The levels of modification were calculated as described in the Materials and methods section, and correspond to mean data with standard deviation from at least three independent experiments carried out on different days.


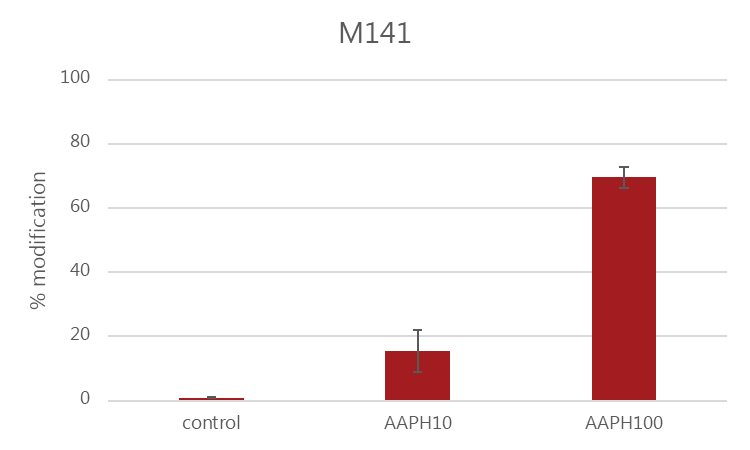

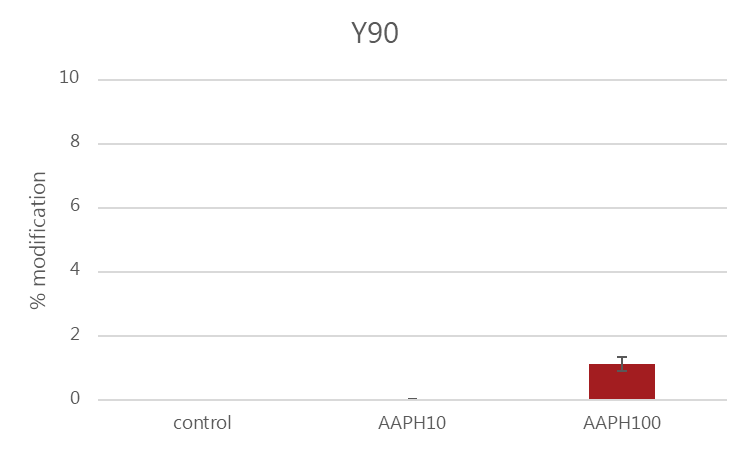

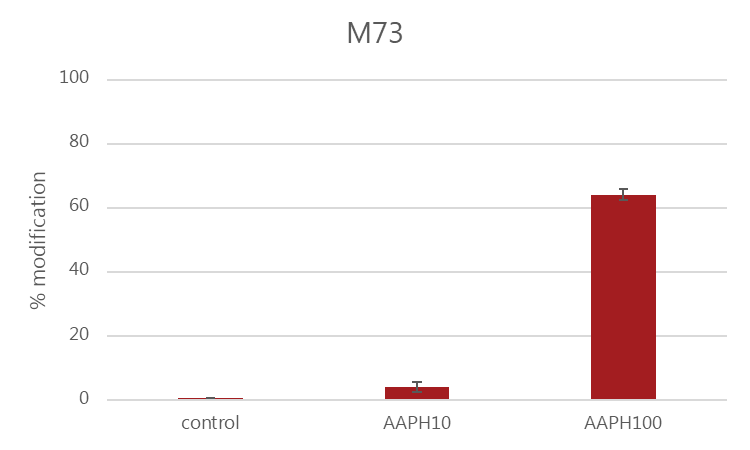

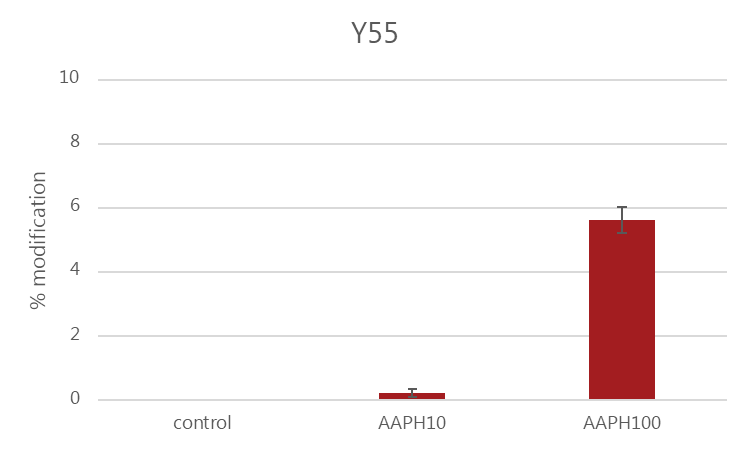

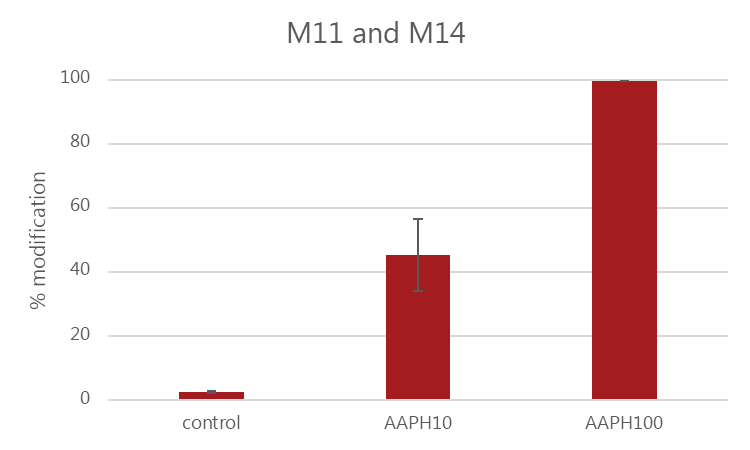

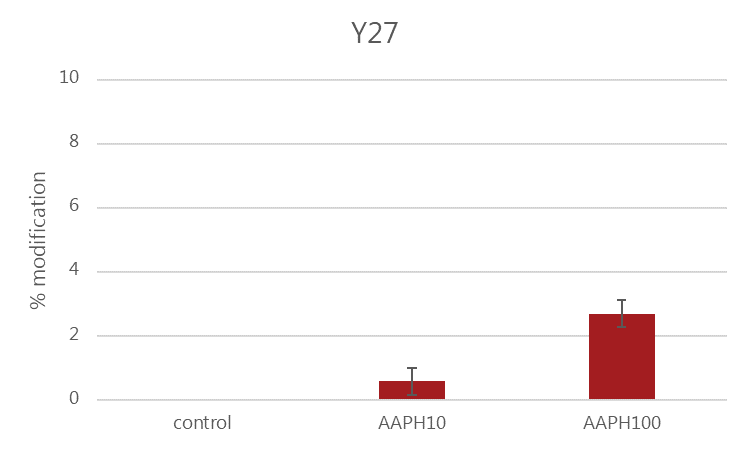


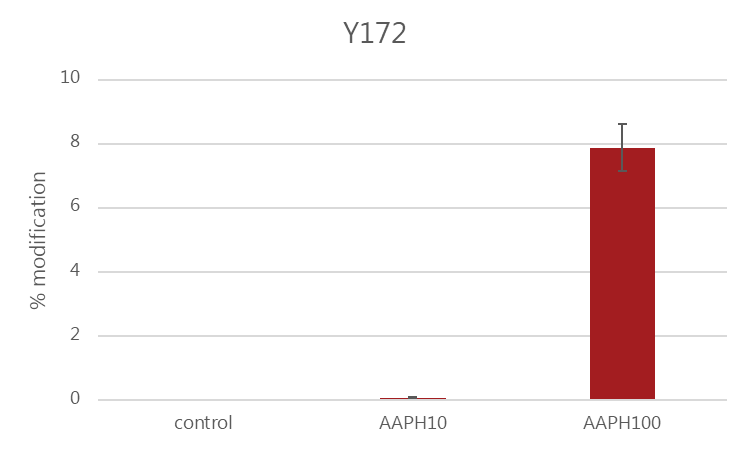

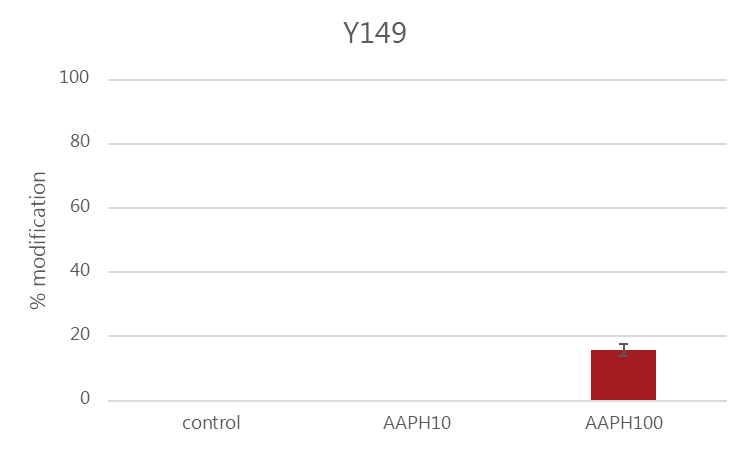


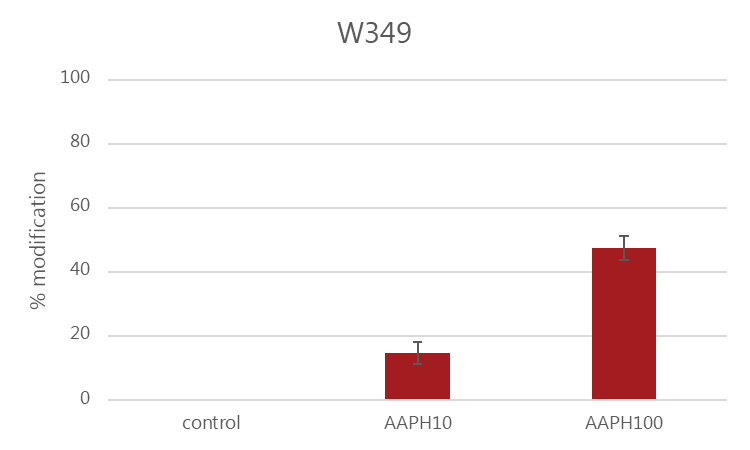

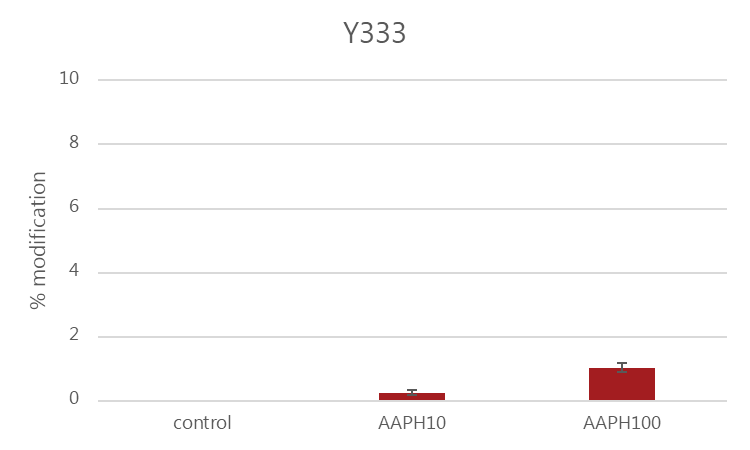

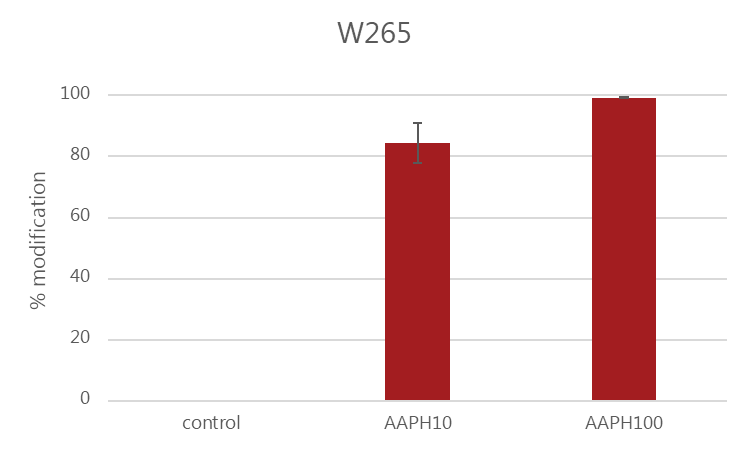

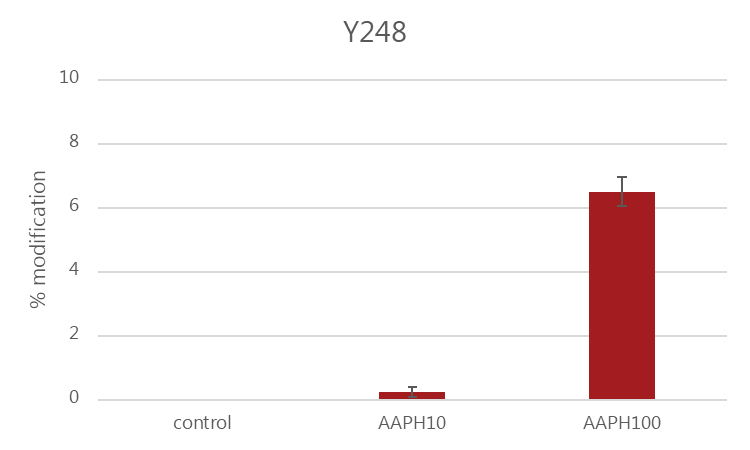

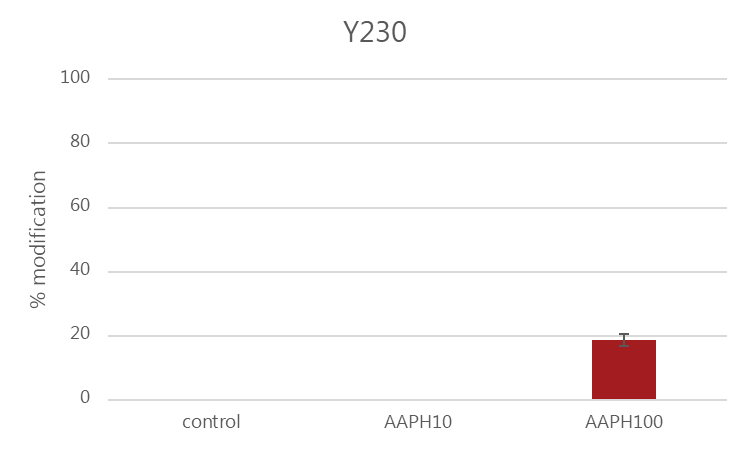

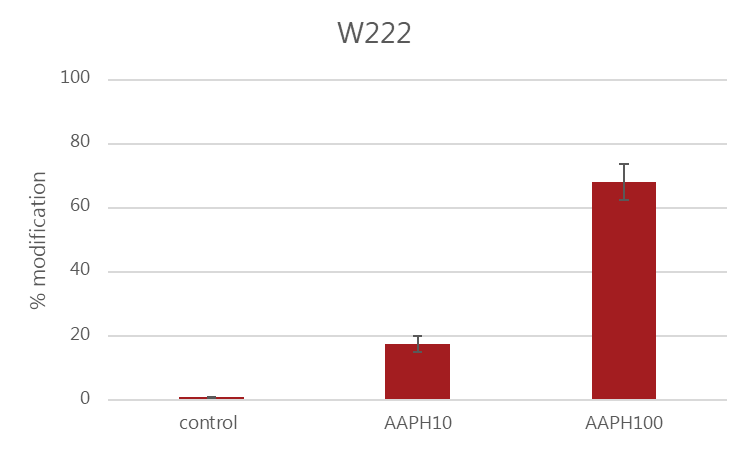

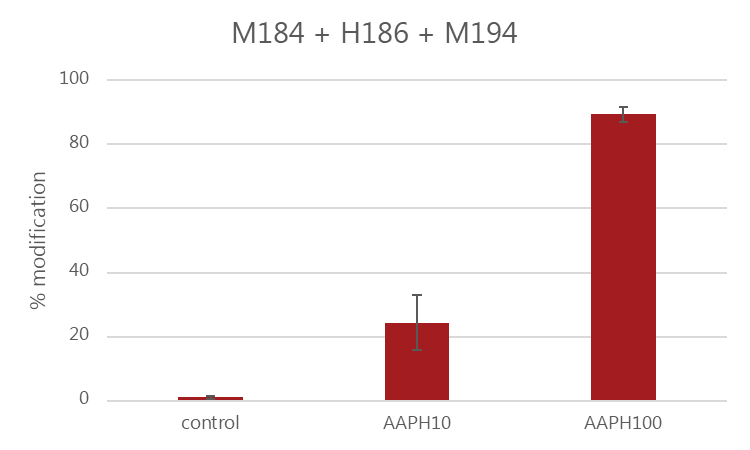

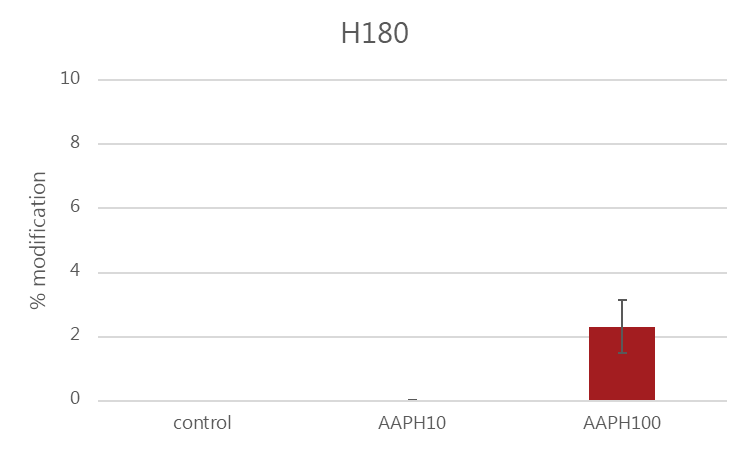


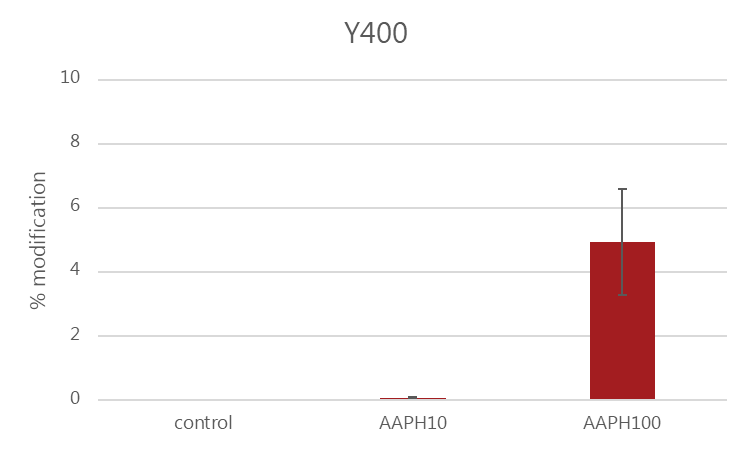

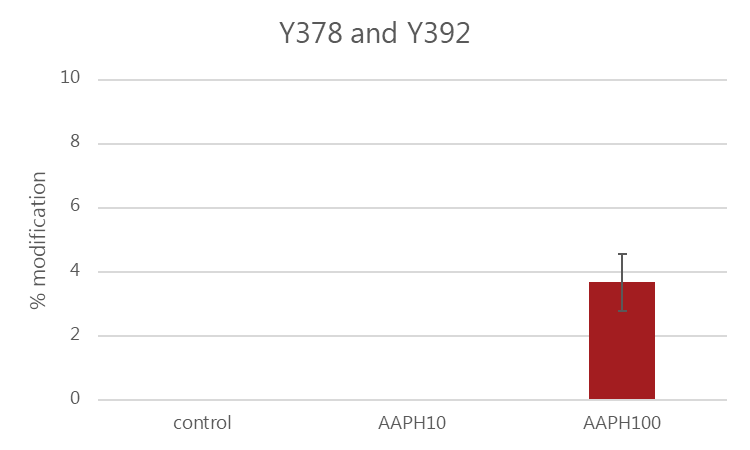

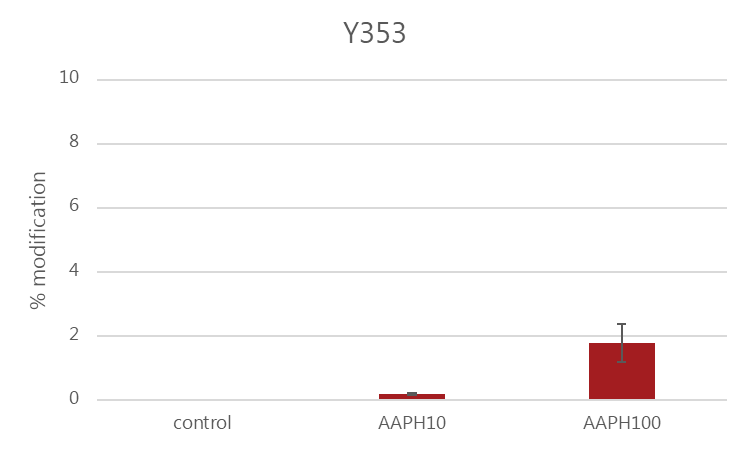


**Figure S6:** Quantification of the % modification of the peptides containing Trp, Met, Tyr and/or His residues obtained after digestion of 6PGDH. The levels of modification were calculated as described in the Materials and methods, and correspond to mean data with standard deviation from at least three independent experiments carried out on different days.


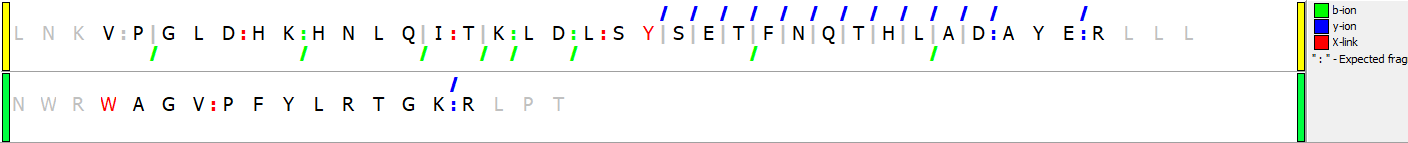


**A**


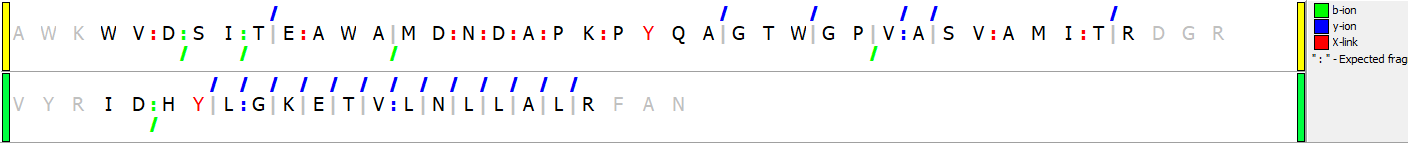


**B**


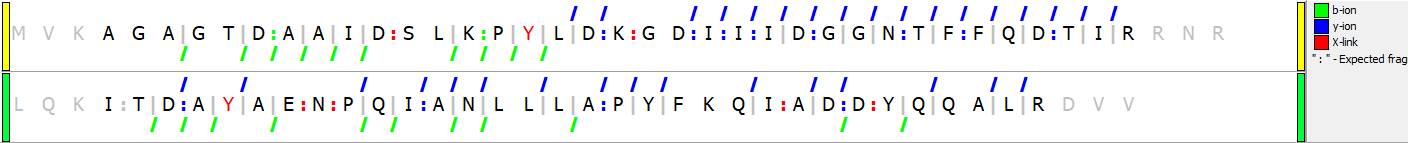


**C**

**Figure S7:** MS/MS of the crosslinks between W328 and Y408 (Trp-Tyr) and Y178–Y466 (di-Tyr) observed after oxidation of G6PDH induced by AAPH-derived ROO^•^ (100 mM, 180 min at 37 °C) (Panels A and B), and between Y90-Y378 (di-Tyr) observed after oxidation of 6PGDH induced by AAPH- ROO^•^ (100 mM, 180 min at 37 °C) (Panel C). Further data on each cross-link is presented in **Table 4**.


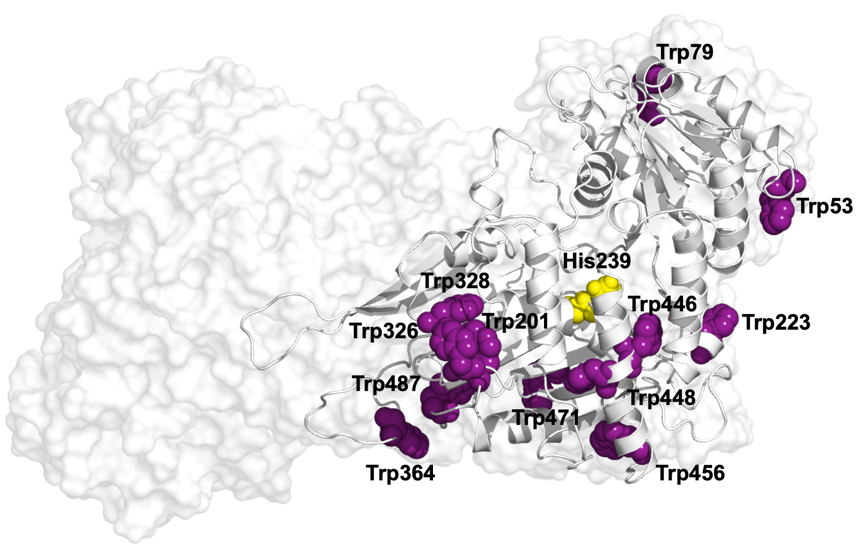

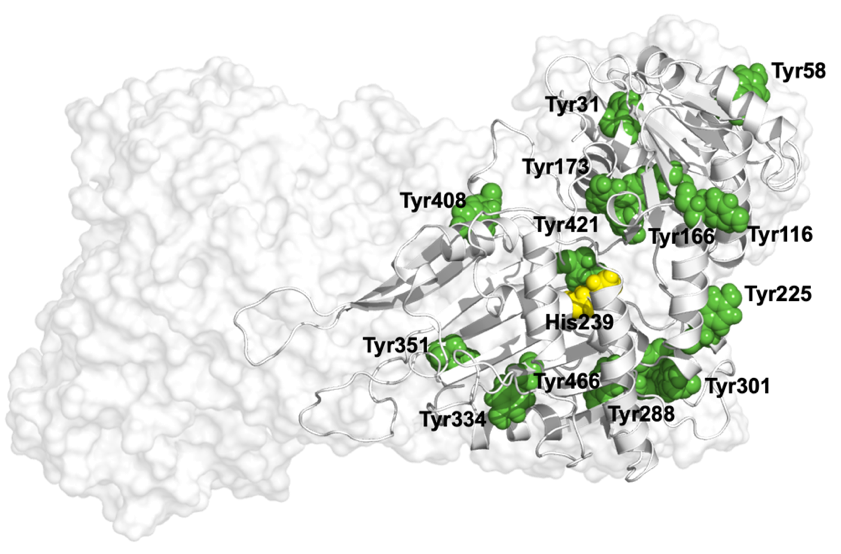


**C**

**B**

**A**

**Figure S8:** Rendering of the locations of Trp (panel A), Met (panel B), and Tyr (panel C) residues in the 3-D structure of G6PDH (built as described in Materials and methods section). Chains of each dimer are presented in light grey and each residue indicated with particular colors and their location indicated. For one of the chains, the active site residue His239 (yellow) is indicated.


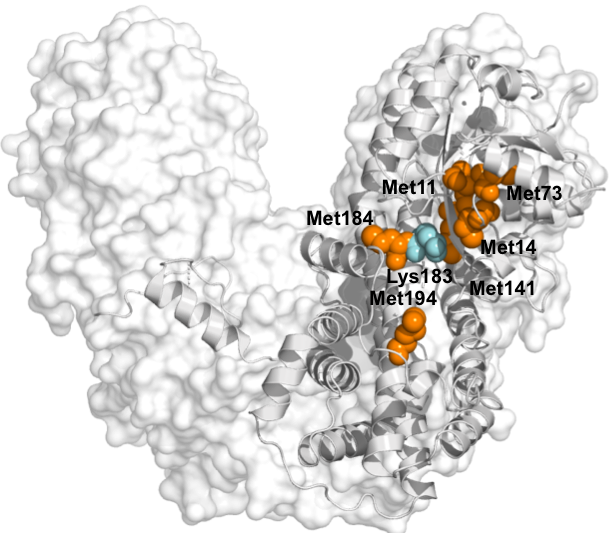


**C**

**B**

**A**


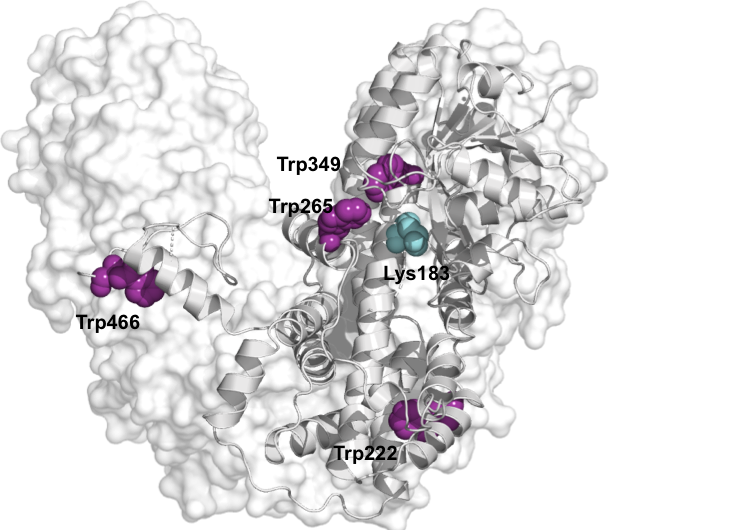

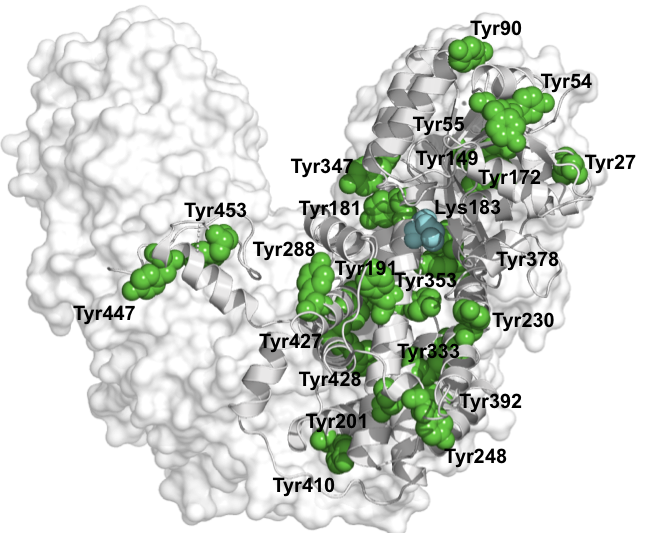


**Figure S9:** Rendering of the locations of Trp (panel A), Met (panel B), and Tyr (panel C) residues in the crystal structure of 6GPDH (PDBid: 2ZYD). Chains of each dimer are presented in light grey and each residue indicated with particular colors and their location indicated. For one of the chains, the active site residue Lys183 (light blue) is indicated.


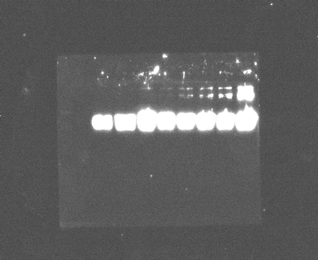

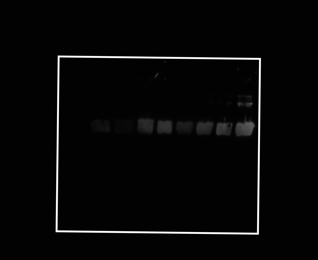

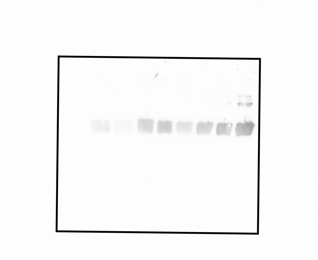

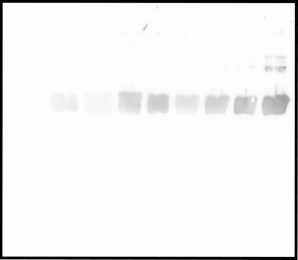


**D.1**

**D.2**

**D.3**

**D.4**

**A.1**

**A.2**

**A.3**

**A.4**

**B.1**

**B.2**

**B.3**

**B.4**

**C.1**

**C.2**

**C.3**

**C.4**

**D.1**

**D.2**

**D.3**


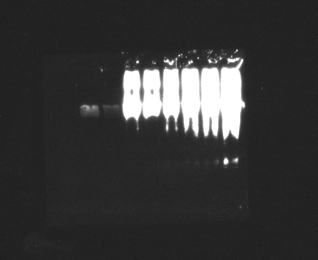

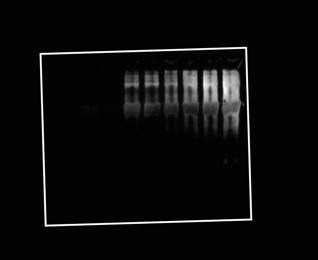

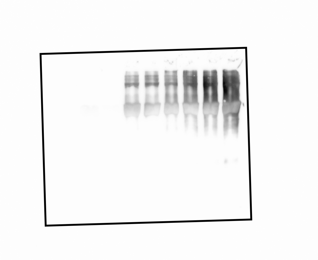

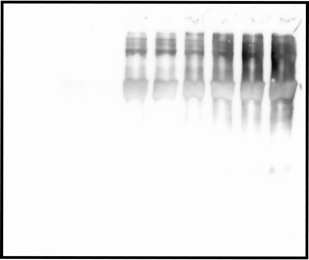


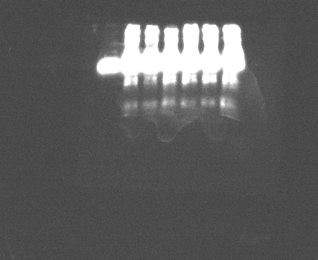

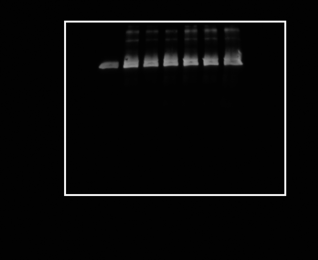

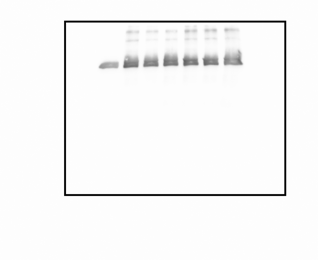

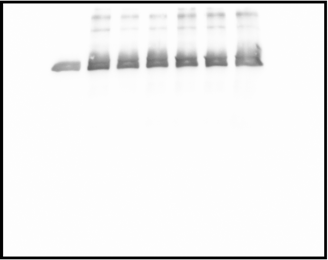


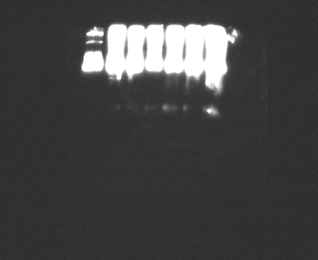

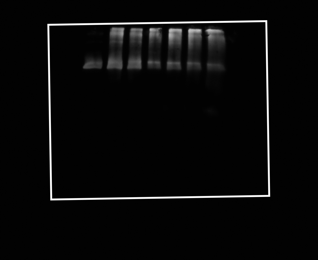

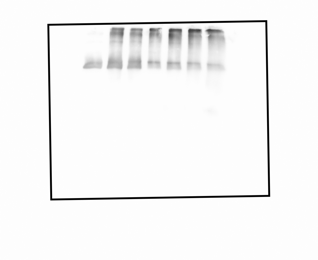

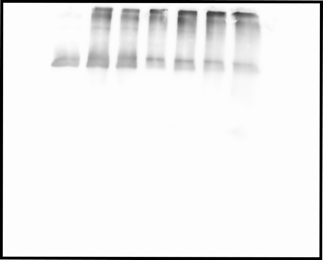


**Figure S10:** Management of images of WB for carbonyls groups. Images of rows A and B correspond to WB of G6PDH obtained during the incubation of the protein with 10 (images A) and 100 (images B) mM AAPH, respectively. Rows C and D show WB images for 6PGDH obtained during its incubation with 10 (images C) and 100 (images D) mM AAPH. Numbers in each set of images (columns) represent the digital image processing; column 1 (A1, B1, etc.) depicts images obtained by modifying dark intensity of original images, which was carried out to identify the edge of membranes. Column 2 (A2, B2, etc.) corresponds to original WB images, obtained with 50 s exposure. Column 3 (A3, B3, etc.) depicts color inversion of the images presented in column 2, while column 4 corresponds to cropped and aligned images of column 3. The latter images were included in the Figure 5 (C4 and D4), or Figure S3 (A4 and B4). To simplify this Figure (Fig. S10), no indication of the experimental condition of each lane was included. Such information is presented in Fig.5 and Fig.S3. Squares in columns 2, 3, and 4 show the limit of each edge of membranes.


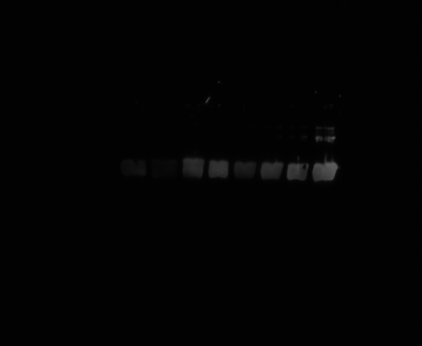

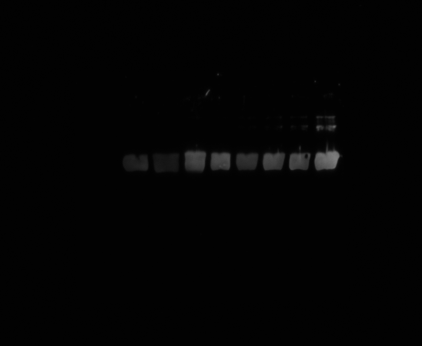

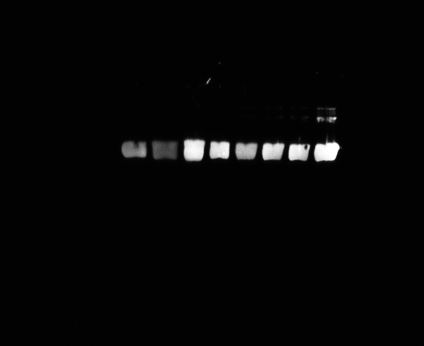


**D.1**

**D.2**

**D.3**

**A.1**

**A.2**

**A.3**

**B.1**

**B.2**

**B.3**

**C.1**

**C.2**

**C.3**


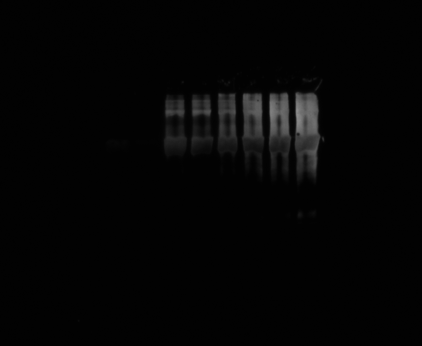

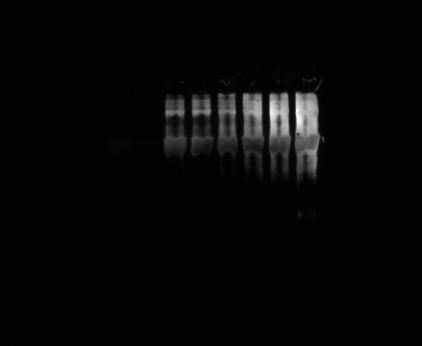

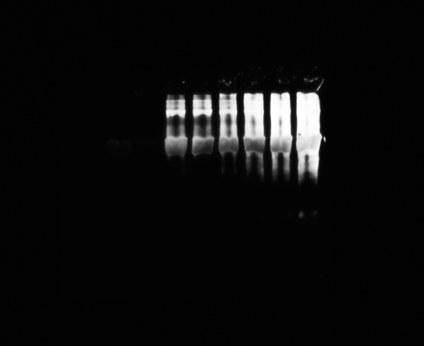


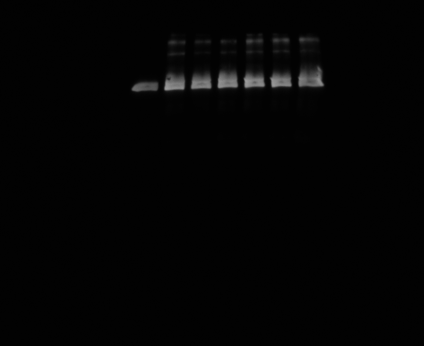

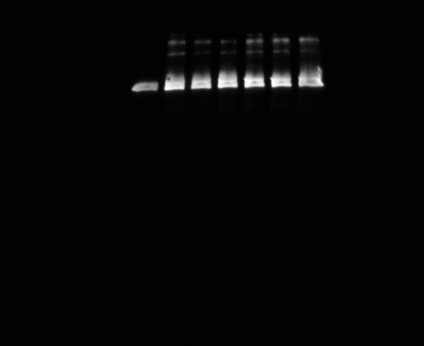

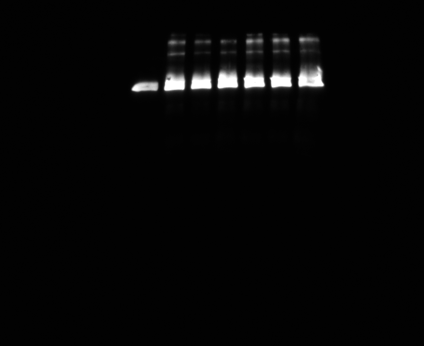


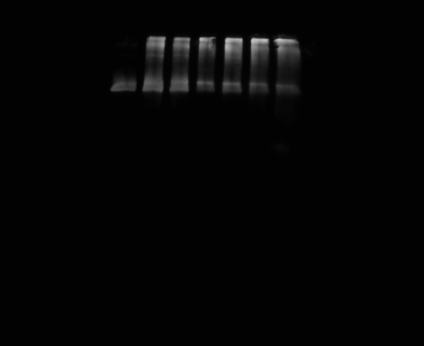

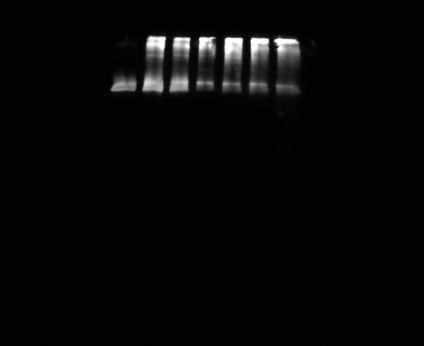

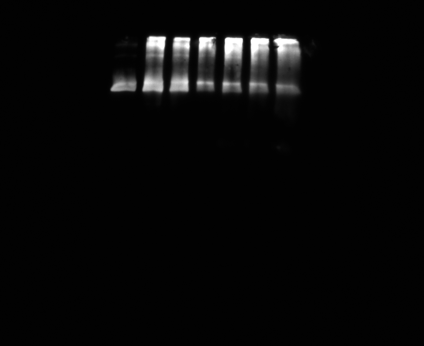


**50 s**

**2 min**

**3 min**

**Figure S11:** Images of WB for carbonyls groups obtained at different (gradual) times of exposure. Images A and B correspond to WB of G6PDH obtained during the incubation of the protein with 10 (images A) and 100 (images B) mM AAPH, respectively. Images C and D show WB for 6PGDH obtained during its incubation with 10 (images C) and 100 (images D) mM AAPH. Numbers in each set of images (columns) represent a particular time of exposure, as indicated at the bottom of the figure. To simplify the figure, no indication about the experimental condition of each lane of gels was included, this information is presented in Fig.5 and Fig.S3.

| **Peptide sequence** | **Mass-to-charge ratio (*m/z*)** | **Mass error (ppm)** | **Modifications detected (Da shift)** |
| --- | --- | --- | --- |
| ADWDKAAYTK | 600.7802 (+2) | -0.1752 | W: +16; +32 |
| ADWDKAAYTKVVR | 508.2719 (+3) | 0.8051 | W: +16; +32 |
| AGQLNPDTR | 486.2489 (+2) | -1.0931 | - |
| CSEVVVYFK | 565.7812 (+2) | -0.5882 | Y: +16 |
| CSEVVVYFKTPELNLFK | 1037.0399 (+2) | -0.0170 | - |
| CSEVVVYFKTPELNLFKESWQDLPQNK | 825.4168 (+4) | 1.2635 | - |
| DEVEEAWK | 503.2298 (+2) | -2.1180 | W446: +4; +16; +32 |
| DMIQNHLLQILCMIAMSPPSDLSADSIR | 1057.1886 (+3) | -0.5379 | M235: +16  H239: +16  M246: +16  M249: +16 |
| DMIQNHLLQILCMIAMSPPSDLSADSIRDEK | 1181.2434 (+3) | -0.2550 | M235: +16  H239: +16  M246: +16  M249: +16 |
| EALETFMK | 484.7415 (+2) | -0.2041 | M70: +16 |
| EALETFMKETIDEGLWDTLSAR | 639.5638 (+4) | 0.1839 | M70: +16  W79: +4; +14; +16; +32 |
| EKTVRGQYTAGFAQGK | 435.9811 (+4) | 0.8291 | Y288: +16 |
| ESWQDLPQNK | 622.7989 (+2) | 0.9597 | W364: +4; +16; +32 |
| ESWQDLPQNKLTIR | 864.4574 (+2) | 1.6339 | W364: +4; +16; +32 |
| ETIDEGLWDTLSAR | 803.3914 (+2) | 0.8879 | W79: +4; +14; +16; +32 |
| ETVLNLLALR | 571.3506 (+2) | 0.5143 | - |
| FANSLFVNNWDNR | 532.9233 (+3) | 0.5837 | W201: +4; +16; +32 |
| GIQALFVR | 452.2742 (+2) | -0.7266 | - |
| GIQALFVRR | 530.3247 (+2) | -0.4017 | - |
| GLGEAKLNAKPAR | 442.2614 (+3) | 0.8255 | - |
| GQYTAGFAQGK | 564.2776 (+2) | -0.1237 | Y288: +16 |
| GQYTAGFAQGKK | 628.3251 (+2) | -0.4680 | Y288: +16 |
| HNLQITK | 427.2481 (+2) | 0.0156 | - |
| IDHYLGK | 423.2294 (+2) | -0.4410 | - |
| IDHYLGKETVLNLLALR | 984.5674 (+2) | -0.7268 | - |
| IDRSNVR | 430.2408 (+2) | 0.7327 | - |
| IDRSNVREKTVR | 736.9182 (+2) | 0.1398 | - |
| IIGVGR | 307.7028 (+2) | 0.2814 | - |
| IIGVGRADWDKAAYTK | 882.4755 (+2) | 0.7324 | W53: +16; +32 |
| ITINYFAMPPSTFGAICK | 508.5074 (+4) | 0.4822 | Y116: +16  M119: +16 |
| ITINYFAMPPSTFGAICKGLGEAK | 862.7745 (+3) | -0.1845 | M119: +16 |
| KLLPSLYQLEK | 444.6026 (+3) | -0.0250 | Y31: +16 |
| KLLPSLYQLEKAGQLNPDTR | 571.8219 (+4) | 0.5385 | Y31: +16 |
| KVPGYLEEEGANK | 478.5805 (+3) | 0.2097 | Y301: +16 |
| KVPGYLEEEGANKSSNTETFVAIR | 660.5882 (+4) | 0.4199 | Y301: +16 |
| LDFCNLDVNDTAAFSR | 929.4254 (+2) | -0.1647 | - |
| LDLSYSETFNQTHLADAYER | 791.7046 (+3) | -0.0113 | Y408: +16  H116: +16 |
| LGAMLDQK | 438.2364 (+2) | 0.4628 | M105: +16; +32 |
| LGAMLDQKNR | 573.3084 (+2) | 0.0692 | M105: +16; +32 |
| LLLETMR | 438.2546 (+2) | -0.9064 | M429: +16; +32 |
| LLLETMRGIQALFVR | 587.3466 (+3) | -0.7848 | M429: +16; +32 |
| LLLETMRGIQALFVRR | 479.7870 (+4) | -0.1666 | M429: +16; +32 |
| LLPSLYQLEK | 602.3528 (+2) | -0.4842 | Y31: +16 |
| LLPSLYQLEKAGQLNPDTR | 719.3952 (+3) | -0.2462 | Y31: +16 |
| LNAKPAR | 385.2376 (+2) | -0.6279 | - |
| LQPDEGVDIQVLNK | 523.2824 (+3) | 0.0632 | - |
| LQPDEGVDIQVLNKVPGLDHK | 579.3155 (+4) | 1.9694 | - |
| LQPDEGVDIQVLNKVPGLDHKHNLQITK | 787.9333 (+4) | 0.2113 | - |
| NRITINYFAMPPSTFGAICK | 767.7221 (+3) | 0.4124 | M119: +16 |
| PYQAGTWGPVASVAMITRDGR | 559.0350 (+4) | 5.1716 | - |
| RDEVEEAWK | 581.2804 (+2) | -0.7558 | W446: +4; +16; +32 |
| RIDRSNVR | 508.2914 (+2) | 0.2519 | - |
| RKLLPSLYQLEK | 372.7290 (+4) | 0.2439 | Y31: +16 |
| RKLLPSLYQLEKAGQLNPDTR | 488.8792 (+5) | 1.2983 | - |
| SLRRIDR | 305.8543 (+3) | 0.2961 | - |
| SSNTETFVAIR | 612.8146 (+2) | 0.1648 | - |
| SSNTETFVAIRVDIDNWR | 1062.0294 (+2) | -0.6082 | W326: +4; +16; +32 |
| TIDHVEITVAEEVGIEGR | 656.3409 (+3) | -0.0693 | - |
| TIDHVEITVAEEVGIEGRWGYFDKAGQMR | 1102.8786 (+3) | -1.3439 | W223: +4; +16; +32 |
| TPELNLFK | 481.2713 (+2) | -0.2450 | - |
| TPELNLFKESWQDLPQNK | 1094.0577 (+2) | -0.2895 | W364: +4; +16; +32 |
| TPELNLFKESWQDLPQNKLTIR | 668.3617 (+4) | 0.4611 | W364: +4; +16; +32 |
| TVRGQYTAGFAQGK | 742.3862 (+2) | 0.9251 | Y288: +16 |
| TVRGQYTAGFAQGKK | 403.7205 (+4) | 3.0020 | Y288: +16 |
| VDIDNWR | 459.2274 (+2) | 0.8501 | W326: +4; +16; +32 |
| VDIDNWRWAGVPFYLR | 669.6799 (+3) | 0.9587 | W326: +4; +16; +32  W328: +4; +14; +16; +32 |
| VLKSLR | 358.2449 (+2) | -2.0991 | - |
| VLKSLRR | 436.2954 (+2) | 0.4711 | - |
| VPGLDHK | 383.2163 (+2) | 0.7649 | - |
| VPGLDHKHNLQITK | 800.4519 (+2) | 0.2293 | - |
| VPGYLEEEGANK | 653.3197 (+2) | -0.7892 | Y301: +16 |
| VPGYLEEEGANKSSNTETFVAIR | 628.5645 (+4) | 0.7021 | Y301: +16 |
| VVMEKPLGTSLATSQEINDQVGEYFEECQVYR | 930.6946 (+4) | -0.2933 | Y166: +16  Y173: +16 |
| VVREALETFMK | 661.8605 (+2) | -1.4701 | M70: +16 |
| VVREALETFMKETIDEGLWDTLSAR | 970.4953 (+3) | 0.5405 | M70: +16  W79: +4; +14; +16; +32 |
| WAGVPFYLR | 554.8005 (+2) | 0.1833 | W328: +4; +14; +16; +32 |
| WGYFDK | 408.1897 (+2) | -1.7759 | W223: +4; +16; +32 |
| WGYFDKAGQMR | 453.5485 (+3) | 0.1389 | - |
| WVDSITEAWAMDNDAPKPYQAGTWGPVASVAMITR | 1278.9467 (+3) | 0.6250 | - |

**Table S1:** List of peptides detected in G6PDH samples with Trp (W), Tyr (Y), His (H) and Met (M) residues underlined. The mass error (ppm) of the unmodified peptides and the modifications detected for W, Y, H and M (+4; +14; +16; +32 Da shift) are indicated.

| **Peptide sequence** | **Mass-to-charge ratio (*m/z*)** | **Mass error (ppm)** | **Modifications detected (Da shift)** |
| --- | --- | --- | --- |
| AASEEYNWDLNYGEIAK | 658.3023 (+3) | 0.4211 | W349: +4; +16; +32  Y353: +16 |
| AASEEYNWDLNYGEIAKIFR | 797.0535 (+3) | -1.5732 | W349: +4; +16; +32  Y353: +16 |
| AAVLPANLIQAQR | 682.9041 (+2) | 0.4210 | - |
| AEFIEK | 368.6974 (+2) | 0.6821 | - |
| AGAGTDAAIDSLKPYLDK | 602.6475 (+3) | -0.4131 | - |
| AGAGTDAAIDSLKPYLDKGDIIIDGGNTFFQDTIR | 917.9666 (+4) | 0.1669 | Y90: +16 |
| AGAGTDAAIDSLKPYLDKGDIIIDGGNTFFQDTIRR | 956.9918 (+4) | 0.9512 | Y90: +16 |
| AGCIIR | 345.1918 (+2) | -1.3888 | - |
| ALYLGKIVSYAQGFSQLR | 672.0422 (+3) | 0.5796 | - |
| AQFLQK | 367.7134 (+2) | -0.3861 | - |
| DEDGNYLVDVILDEAANK | 664.9847 (+3) | -0.1928 | - |
| DEDGNYLVDVILDEAANKGTGK | 779.3799 (+3) | -0.3819 | - |
| DIFTKK | 376.2211 (+2) | 0.4570 | - |
| DIFTKKDEDGNYLVDVILDEAANK | 909.1237 (+3) | -0.3398 | Y248: +16 |
| DIFTKKDEDGNYLVDVILDEAANKGTGK | 614.5143 (+5) | 0.6223 | - |
| DVVAYAVQNGIPVPTFSAAVAYYDSYR | 1468.7269 (+2) | 0.8545 | - |
| DYFGAHTYK | 551.2536 (+2) | 2.0703 | - |
| DYFGAHTYKR | 629.3042 (+2) | -1.0345 | - |
| EAYELVAPILTK | 449.5904 (+3) | 0.2357 | Y149: +14; +16 |
| EFVESLETPR | 603.8037 (+2) | 0.7134 | - |
| EFVESLETPRR | 454.9053 (+3) | -0.1557 | - |
| EKTEEVIAENPGK | 481.9157 (+3) | 0.1633 | - |
| EKTEEVIAENPGKK | 524.6140 (+3) | 0.2667 | - |
| ELSAEGFNFIGTGVSGGEEGALK | 757.0376 (+3) | -0.2067 | - |
| ELSAEGFNFIGTGVSGGEEGALKGPSIMPGGQK | 1074.5309 (+3) | 0.3826 | M141: +16 |
| GDIIIDGGNTFFQDTIR | 941.4707 (+2) | -0.7009 | - |
| GDIIIDGGNTFFQDTIRR | 680.0166 (+3) | -0.2531 | - |
| GGLNLTNEELAQTFTEWNNGELSSYLIDITK | 868.4295 (+4) | 0.7759 | W222: +4; +16; +32  Y230: +16 |
| GPSIMPGGQK | 486.2526 (+2) | 0.3769 | M141: +16 |
| GPSIMPGGQKEAYELVAPILTK | 1150.1219 (+2) | -0.7225 | M141: +16  Y149: +16 |
| GTGKWTSQSALDLGEPLSLITESVFAR | 955.1661 (+3) | 0.1466 | W265: +4; +16; +32 |
| GYTVSIFNR | 528.7773 (+2) | 0.3887 | Y27: +16 |
| GYTVSIFNRSREKTEEVIAENPGK | 908.80323 (+3) | -0.2553 | - |
| IDKEGVFHTEWLD | 794.8857 (+2) | -0.1250 | - |
| ILLMVK | 358.7406 (+2) | -0.3360 | M73: +16 |
| ITDAYAENPQIANLLLAPYFK | 789.0861 (+3) | -0.8410 | Y378: +16  Y392: +16 |
| IVSYAQGFSQLR | 684.8672 (+2) | -0.3068 | Y333: +16 |
| IAAVAEDGEPCVTYIGADGAGHYVK | 855.0760 (+3) | 0.2109 | Y172: +16  H180: +16 |
| KDEDGNYLVDVILDEAANK | 707.6831 (+3) | -0.2476 | Y248: +16 |
| KDEDGNYLVDVILDEAANKGTGK | 822.0782 (+3) | -0.5036 | Y248: +16 |
| KLVPYYTVK | 555.8315 (+2) | 0.1216 | Y55: +16 |
| LVPYYTVK | 491.784036 (+2) | 0.4992 | Y55: +16 |
| LVPYYTVKEFVESLETPR | 724.0525 (+3) | -0.4084 | - |
| LVPYYTVKEFVESLETPRR | 582.3165 (+4) | 0.6110 | - |
| MVHNGIEYGDMQLIAEAYSLLK | 1248.1191 (+2) | 1.6300 | M184: +16  H186: +16  M194: +16 |
| NLALNIESR | 515.2880 (+2) | 0,1771 | - |
| NRELSAEGFNFIGTGVSGGEEGALK | 847.0856 (+3) | -0.2062 | - |
| NRELSAEGFNFIGTGVSGGEEGALKGPSIMPGGQK | 1164.5789 (+3) | 0.3278 | M141: +16 |
| QIADDYQQALR | 660.8308 (+2) | -0.3735 | Y400: +16 |
| QIADDYQQALRDVVAYAVQNGIPVPTFSAAVAYYDSYR | 1060.2762 (+4) | 0.1990 | Y400: +16 |
| QQIGVVGMAVMGR | 673.3576 (+2) | 0.3534 | M11: +16  M14: +16 |
| RALYLGK | 410.7556 (+2) | -0.2015 | - |
| RILLMVK | 436.7911 (+2) | -0.3390 | M73: +16 |
| RIDKEGVFHTEWLD | 582.2933 (+3) | 1.4293 | - |
| SKQQIGVVGMAVMGR | 520.9498 (+3) | -2.2213 | M11: +16  M14: +16 |
| SREKTEEVIAENPGKK | 605.6584 (+3) | 0.6749 | - |
| TEEVIAENPGK | 593.8011 (+2) | 1.6150 | - |
| VLSGPQAQPAGDK | 634.3357 (+2) | 1.3870 | - |
| VLSGPQAQPAGDKAEFIEK | 993.0205 (+2) | 0.8705 | - |
| VAASKVLSGPQAQPAGDK | 862.4705 (+2) | 0.0602 | - |
| VAASKVLSGPQAQPAGDKAEFIEK | 611.0813 (+4) | 0,90636 | - |
| WTSQSALDLGEPLSLITESVFAR | 840.7709 (+3) | -1.5260 | W265: +4; +16; +32 |
| YISSLK | 355.7078 (+2) | -0.5372 | - |
| YISSLKDQR | 555.3011 (+2) | -0.5558 | - |
| YISSLKDQRVAASK | 783.4359 (+2) | -0.5552 | - |

**Table S2:** List of peptides detected in 6PGDH samples with Trp (W), Tyr (Y), His (H) and Met (M) residues underlined. The mass error (ppm) of the unmodified peptides and the modifications detected for W, Y, H and M (+4; +14; +16; +32 Da shift) are indicated.
